# Effects of repetitive transcranial magnetic stimulation on upper limb motor recovery after stroke: an overview of systematic reviews

**DOI:** 10.3389/fneur.2026.1797218

**Published:** 2026-06-10

**Authors:** Linli Zhang, Chengshuo Wang, Zujian Zhang, Hongwei Sun

**Affiliations:** 1 Sports Science and Technology, Guangzhou College of Applied Science and Technology School, Guangzhou, China; 2 Urban and Rural Cultural Development Research Center, Guangdong Provincial Social Science Research Base, Guangzhou, China; 3 School of Exercise and Health, Shanghai University of Sport, Shanghai, China; 4 School of Clinical Medicine, Youjiang Medical University for Nationalities, Baise, China

**Keywords:** stroke, repetitive transcranial magnetic stimulation, upper limb, motor function, overview of systematic reviews, radar plot

## Abstract

**Background:**

Well-conducted meta-analyses and systematic reviews (MAs/SRs) provide high-level evidence to inform clinical decision-making. However, the overall methodological rigor and reporting quality of MAs/SRs evaluating the effects of repetitive transcranial magnetic stimulation (rTMS) on upper limb motor recovery after stroke have not yet been comprehensively assessed. Therefore, this study aimed to critically appraise the methodological and reporting quality of existing systematic reviews and meta-analyses addressing this topic.

**Methods:**

A systematic search was conducted across nine electronic databases (CNKI, Wanfang, VIP, CBM, PubMed, Embase, Web of Science, Cochrane Library, and ClinicalTrials.gov) and gray literature from inception to April 1, 2024. Two reviewers independently performed literature screening and data extraction. Methodological quality and risk of bias were assessed across six dimensions—including publication year, homogeneity, study type, publication bias, AMSTAR-2 score, and PRISMA 2020 score—using AMSTAR-2 and PRISMA 2020 criteria, visualized via radar plots. Finally, the certainty of evidence for outcomes was graded using the Grading of Recommendations Assessment, Development and Evaluation (GRADE) approach.

**Results:**

A total of 17 MAs/SRs were included, with an average multivariate rank score of 11.82. GRADE assessment revealed that the certainty of evidence was high for 13 outcome indicators, moderate for 47, low for 91, and very low for 71. Overall, the methodological and reporting quality was suboptimal. Insufficient methodological rigor and incomplete reporting were identified as the primary drivers of downgraded evidence, while publication year and potential bias played secondary roles.

**Conclusion:**

Although current systematic reviews suggest that rTMS may enhance upper limb motor function post-stroke, the overall quality of evidence remains limited, and its impact on muscle spasticity warrants further investigation. Future research must adopt rigorous methodological standards and transparent reporting practices. Crucially, integrating MRI-derived biomarkers is needed to address current evidence heterogeneity, ultimately facilitating the transition toward personalized, biomarker-driven neuromodulation in stroke rehabilitation.

**Systematic review registration:**

www.crd.york.ac.uk/prospero/, identifier, CRD42024523846.

## 1 Introduction

Stroke remains a leading cause of long-term disability worldwide, imposing a substantial public health burden (1). Globally, over 80 million individuals live with the sequelae of stroke, with 70–90% suffering from persistent upper limb motor impairment (2). Despite advancements in rehabilitation, complete functional recovery remains elusive for most; hand function recovery is particularly suboptimal, with only 12% of patients achieving satisfactory functional outcomes (3). The upper limb is a cornerstone of human functional independence, accounting for an estimated 60% of total body function, with the hand alone facilitating nearly 90% of these intricate activities. Unlike the lower limbs, which primarily serve locomotor purposes, the upper extremity requires a sophisticated synergy of fine motor control and complex coordination. Consequently, the restoration of upper limb and hand function remains a significant bottleneck in stroke rehabilitation, profoundly limiting patients’ activities of daily living (ADL) (4, 5). While systematic reviews confirm the efficacy of diverse rehabilitation paradigms (6-8), traditional “bottom-up” approaches—such as Brunnstrom, Rood, and Bobath therapies—face inherent physiological constraints. These neurodevelopmental techniques aim to harness neural plasticity indirectly by modulating sensory input and reinforcing peripheral movement patterns (9-11). However, their clinical utility is often hampered by high resource intensity, a heavy reliance on patient cognition and volition, and diminishing returns in cases of severe impairment (12). As survivors’ aspirations shift toward societal reintegration and higher quality of life, relying solely on peripheral interventions has proven insufficient. There is, therefore, an imperative need to explore “top-down” neuromodulatory interventions that directly target the central nervous system, aiming to accelerate functional recovery, restore self-care autonomy, and ultimately mitigate the socioeconomic burden of stroke.

Neural plasticity underlies motor relearning and functional recovery after stroke (13). Well-timed, high-intensity sensorimotor input promotes perilesional cortical reorganization and axonal sprouting, enabling compensatory pathways that bypass damaged circuits and restore coordinated cortical network activity (14). Building on these principles, repetitive transcranial magnetic stimulation (rTMS) has become a leading non-invasive neuromodulatory technique in post-stroke motor rehabilitation (15). Through electromagnetic induction, rTMS generates focal currents in the cerebral cortex, allowing precise modulation of cortical excitability. It can either enhance ipsilesional excitability or suppress maladaptive contralesional inhibition, thereby rebalancing interhemispheric activity (16, 17). rTMS protocols are typically classified as excitatory or inhibitory (18). High-frequency rTMS (> 1 Hz) and intermittent theta-burst stimulation (iTBS) increase cortical excitability, while low-frequency rTMS (≤ 1 Hz) and continuous theta-burst stimulation (cTBS) reduce excessive activity (19, 20). Repeated stimulation can induce synaptic plasticity resembling long-term potentiation (LTP) or long-term depression (LTD), with effects propagating across distributed motor networks to ultimately support recovery of skilled upper-limb function (21).

Repetitive transcranial magnetic stimulation has gained significant traction as a therapeutic cornerstone in post-stroke upper limb rehabilitation. Although numerous primary studies confirm its efficacy in improving muscle strength and fine motor coordination, the rapid proliferation of MAs/SRs has introduced a critical challenge: methodological heterogeneity. The reliability of these high-level syntheses is often undermined by subjective interpretations and inconsistent application of assessment tools, introducing substantial variability into study designs (22). Given that clinical decision-making depends heavily on the integrity of synthesized evidence, low-quality MAs/SRs risk misleading clinical practice. A rigorous, standardized quality appraisal of existing MAs/SRs on rTMS for upper limb recovery post-stroke is, therefore, both timely and essential. To date, this critical evaluative gap remains unaddressed in the literature.

An overview of systematic reviews serves to synthesize and critically appraise the quality of existing MAs/SRs, providing a robust evidence base to inform clinical decision-making (23). In this study, radar plots were employed to visually represent six key dimensions: publication year, study type, homogeneity, and publication bias, together with quantitative evaluations based on the AMSTAR-2 and PRISMA 2020 checklists (24, 25). Furthermore, the Grading of Recommendations Assessment, Development and Evaluation (GRADE) approach (26) was utilized to determine the certainty of evidence for the reported outcome indicators. This study aims to offer a comprehensive, visual synthesis of the current evidence landscape, thereby providing a clear and reliable framework to guide the clinical implementation of rTMS in post-stroke upper limb recovery.

## 2 Materials and methods

### 2.1 Study enrollment and reporting

This overview of systematic reviews was conducted in accordance with the Cochrane Handbook for Systematic Reviews of Interventions (27) and reported according to the Preferred Reporting Items for Overviews of Reviews (PRIOR) guideline (28). The protocol was prospectively registered in the International Prospective Register of Systematic Reviews (PROSPERO; registration number: CRD42024523846, registered on 23 March 2024).

### 2.2 Search strategy

Two reviewers (LLZ and ZJZ) independently conducted a comprehensive literature search across eight electronic databases from inception to April 1, 2024, including PubMed, Embase, Web of Science, the Cochrane Database of Systematic Reviews, and four Chinese databases (CNKI, VIP, Wanfang, and CBM). To capture ongoing, unpublished, and gray literature, we systematically searched ClinicalTrials.gov, the Gray Matters checklist, and OpenGrey.^1^ In addition, reference lists of eligible studies, relevant conference proceedings, and the PROSPERO database were manually screened to identify further records. The search strategy combined Medical Subject Headings (MeSH) and free-text terms—including “stroke,” “repetitive transcranial magnetic stimulation,” and “upper limb”—using appropriate Boolean operators (AND/OR). Detailed search strings for all databases are provided in Supplementary Tables 1, 2. The study selection process is illustrated in a PRISMA flow diagram (29).

### 2.3 Eligibility criteria

To ensure a systematic and unbiased selection process, eligibility criteria were defined *a priori* based on the PICOS (Population, Intervention, Comparison, Outcome, and Study design) framework (31).

#### 2.3.1 Inclusion criteria

(1) Population (P): Adults (≥ 18 years) with a clinical diagnosis of ischemic or hemorrhagic stroke presenting with upper limb (UL) motor impairment were eligible for inclusion. To ensure a comprehensive evidence synthesis, no restrictions were imposed regarding sex, stroke phase (acute, subacute, or chronic), lesion location (cortical or subcortical), or baseline stroke severity.
(2) Intervention (I): Eligible interventions comprised various repetitive transcranial magnetic stimulation (rTMS) protocols, specifically high-frequency rTMS and theta-burst stimulation (both intermittent and continuous). These neuromodulatory techniques could be administered either as a monotherapy or as an adjuvant to conventional rehabilitation. The latter encompasses a spectrum of standard-of-care stroke therapies, including but not limited to task-oriented training, constraint-induced movement therapy (CIMT), robot-assisted training, virtual reality (VR) integration, and neuromuscular electrical stimulation.
(3) Comparator (C): Control groups consisted of: (i) sham rTMS (placebo control); (ii) conventional stroke rehabilitation alone (standard-of-care); or (iii) other active non-invasive brain stimulation (NIBS) modalities, such as transcranial direct current stimulation (tDCS). These comparators could be administered either in isolation or as a base for adjunctive therapies, provided they allowed for a direct comparison with the rTMS protocols.
(4) Outcomes (O): Primary outcome: The primary outcome is upper limb motor function, primarily quantified by the Fugl-Meyer Assessment-Upper Extremity (FMA-UE), the Brunnstrom assessment of hand motor function, or Reaching Time (RT). Secondary outcomes: To provide a multi-dimensional synthesis of recovery, secondary indicators are categorized into the following domains: (i) Hand Dexterity and Fine Motor Coordination: Assessed via the Action Research Arm Test (ARAT), Wolf Motor Function Test (WMFT), Box and Block Test (BBT), Purdue Pegboard Test (PPT), Nine-Hole Peg Test (NHPT), Jebsen-Taylor Test (JTT), Finger Tapping (FT), or Keyboard Tapping (KT). (ii) Musculoskeletal Status: Including hand strength (Grip Strength [GS] and Pinch Force [PF]) and upper limb spasticity as measured by the Modified Ashworth Scale (MAS). (iii) Neurophysiological Markers of Cortical Excitability: Quantified through transcranial magnetic stimulation (TMS) parameters, including Motor Evoked Potential (MEP) amplitude and latency, as well as resting, active, and general Motor Thresholds (rMT, aMT, and MT). (iv) Functional Independence and Clinical Severity: Evaluated by Activities of Daily Living (ADL) scales (Barthel Index [BI] or Modified Barthel Index [MBI]), global disability via the modified Rankin Scale (mRS), and neurological deficit severity using the National Institutes of Health Stroke Scale (NIHSS). (v) Safety Profiles: Documented as the incidence and nature of all reported adverse events.
(5) Study design (S): This overview is restricted to peer-reviewed MAs/SRs—including network meta-analyses—that evaluate the clinical efficacy of rTMS for upper limb motor recovery following stroke. To ensure a comprehensive yet rigorous synthesis, only studies published in English or Chinese were eligible for inclusion. Protocols, conference abstracts, and scoping reviews were excluded to maintain the high evidentiary standard of this overview.

#### 2.3.2 Exclusion criteria

Studies were excluded if they met any of the following criteria:

(1) Upper limb motor dysfunction resulting from neurological conditions other than stroke (e.g., cerebral palsy, traumatic brain injury, or Parkinson’s disease).
(2) Duplicate publications or overlapping data from the same study population.
(3) Articles for which the full text was unavailable or complete data could not be obtained after contacting the corresponding authors.
(4) Systematic reviews or meta-analyses that compared two or more different rTMS protocols against each other.
(5) Conference abstracts, study protocols, or non-peer-reviewed publications.
(6) Reviews lacking any outcome measures related to upper limb motor function.
(7) Reviews in which rTMS was combined with other non-invasive brain stimulation techniques (e.g., transcranial direct current stimulation, tDCS).
(8) Systematic reviews or meta-analyses that included fewer than five primary studies.
(9) Studies in which the control group also received active rTMS.

### 2.4 Study selection

Two reviewers (LLZ and ZJZ) independently conducted the study selection process. After removing duplicate records via EndNote 20, they performed an initial screening of titles and abstracts based on the predefined eligibility criteria. Subsequently, the full texts of all potentially eligible studies were retrieved and meticulously appraised for final inclusion. Any discrepancies between the two primary reviewers were resolved by consensus or through consultation with a third independent reviewer (CSW).

### 2.5 Data extraction and quality evaluation

Two independent reviewers (LLZ and ZJZ) extracted data from all included MAs/SRs using a pre-designed, pilot-tested form. For studies that reported both upper and lower limb outcomes, only data related to upper limb motor function were extracted. Any disagreements were resolved by discussion or consultation with a third reviewer (CSW). When data were missing or unclear, the corresponding authors were contacted for clarification or additional information. Data were managed in Microsoft Excel 2016. The following information was collected: (i) General characteristics: country of the corresponding author, publication year, type of review, number of included studies, total sample size, intervention details, outcome measures, conflicts of interest, and funding sources. (ii) Quality-related items: homogeneity of results, assessment of publication bias, AMSTAR-2 score, PRISMA 2020 score, and GRADE certainty ratings.

The methodological quality of the included MAs/SRs was appraised using the AMSTAR-2 tool (30). This instrument comprises 16 items, of which seven are critical (items 2, 4, 7, 9, 11, 13, and 15). Each item was rated as “yes,” “partial yes,” or “no.” For quantitative analysis, a score of 1 was assigned for “yes,” 0.5 for “partial yes,” and 0 for “no,” yielding a maximum total score of 16. Refer to Table 1 for the AMSTAR-2 quality grading criteria. Reporting quality was evaluated using the PRISMA 2020 checklist (31), which consists of 27 items. Each item was scored as 1 (fully reported), 0.5 (partially reported), or 0 (not reported), with a maximum possible score of 27. The reporting rate for each item was calculated as the proportion of reviews achieving “fully reported” or “partially reported” status. Items with a reporting rate below 50% were classified as inadequately reported. Two independent reviewers (LLZ and ZJZ) performed all quality assessments. Any disagreements were resolved through discussion with a third reviewer (CSW).

**Table 1:** AMSTAR-2 quality grading criteria.

Quality grade	Grading criteria
High	No or one non-critical item fails to meet the criterion.
Moderate	More than one non-critical item fails to meet the criterion.
Low	One critical item fails to meet the criterion, with or without non-critical failures.
Very low	More than one critical item fails to meet the criterion, with or without non-critical failures.

At the moderate grade, if multiple non-critical items are not met, the overall quality may be downgraded to low.

### 2.6 Multidimensional evaluation and radar plot construction

Six evaluation dimensions were assessed using a combined qualitative and quantitative approach. Publication year, study type, homogeneity, and publication bias were evaluated qualitatively based on principles of clinical epidemiology and evidence-based medicine (32). Specifically: (i) More recent publication years were ranked higher. (ii) Randomized controlled trials (RCTs) as the primary study type received the highest rank. (iii) Homogeneity was considered high when more than 50% of the outcome indicators across included MAs/SRs showed *p* ≥ 0.10 and I^2^ ≤ 50%; otherwise, low homogeneity was assigned. (iv) Publication bias was judged low if a funnel plot or equivalent statistical test was performed and reported; absence of such assessment resulted in a high-bias ranking. AMSTAR-2 and PRISMA 2020 scores were evaluated quantitatively. For AMSTAR-2, each of the 16 items was scored as 1 (yes), 0.5 (partial yes), or 0 (no), with seven items designated as critical. For PRISMA 2020, each of the 27 items was scored as 1 (fully reported), 0.5 (partially reported), or 0 (not reported). To enable visualization, all six dimensions were converted to a common ranking scale (where the highest possible rank equaled the total number of included MAs/SRs). The average rank score across all dimensions was then calculated. Radar plots were generated and optimized using Microsoft Excel 2016 and Adobe Illustrator CS6 (33).

### 2.7 Evaluation of the quality of evidence

The certainty of evidence for each outcome was appraised using the GRADE approach. The evidence was subject to downgrading based on five domains: risk of bias, inconsistency, indirectness, imprecision, and publication bias. Conversely, three factors were considered for potential upgrading: large magnitude of effect, dose–response gradient, and the presence of residual confounding. Accordingly, the certainty of evidence was categorized into four levels: “high,” “moderate,” “low,” or “very low.”

## 3 Results

### 3.1 Literature screening process and results

**Figure 1:**
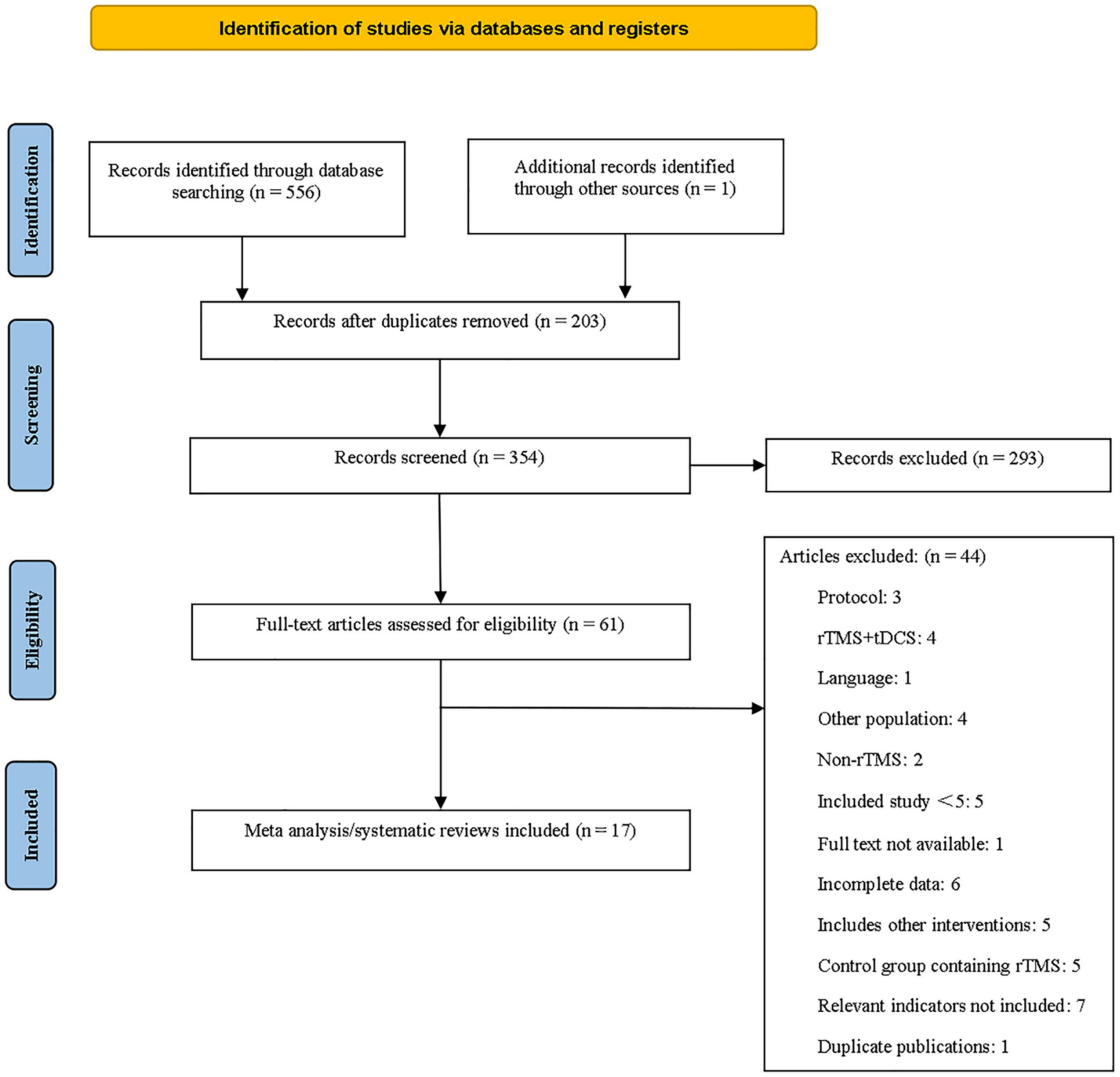
PRISMA flow chart. *n*, number of publications.

The initial search yielded 557 records, including one additional record identified through manual search/reference tracking. After removing 203 duplicate records using EndNote 20, 354 records remained for title and abstract screening. Of these, 293 were excluded, leaving 61 articles for full-text review. Following full-text assessment, 44 articles were excluded, and 17 MAs/SRs were ultimately included in the analysis. The study selection process is illustrated in Figure 1. Supplementary Table 3 provides the list of included and excluded studies and the reasons for exclusion.

### 3.2 Characteristics of the included studies

A total of 17 MAs/SRs were included in this overview (35–51), published between 2012 and 2024. Among these studies, 5 (47–49) were published in Chinese and 12 (35–46) in English. Sixteen were journal articles and one was a master’s thesis (51). Most studies were conducted in mainland China (*n* = 14), with one study conducted in China-Taiwan (40), one in Jordan (35), and one in the Netherlands (44). Regarding methodological quality assessment, nine studies (35, 38, 39, 41, 44, 45, 48, 50, 51) used the Cochrane Risk of Bias tool, five (36, 37, 42, 43, 47) used the Physiotherapy Evidence Database (PEDro) scale, two (40, 46) applied a modified quality assessment checklist, and one study (49) used both the Cochrane Risk of Bias tool and the Newcastle–Ottawa Scale (NOS). Ten studies (35–39, 41, 43–46) explicitly reported no conflicts of interest, whereas seven (40, 42, 47–51) did not provide a statement regarding conflicts of interest. With respect to funding sources, 10 studies (36–38, 40, 43–46, 49, 50) reported funding from non-industrial sources, six (35, 39, 42, 47, 48, 51) did not disclose their funding sources, and one study (41) reported receiving no external funding. Regarding study characteristics, one study (37) focused exclusively on patients with chronic stroke, whereas the remaining studies did not specify stroke duration. One study (48) included only patients with ischemic stroke, while the others included mixed stroke types. Only one study (35) did not perform a meta-analysis. Detailed characteristics of the included studies are presented in Table 2 and Supplementary Table 4.

**Table 2:** The fundamental characteristics of MAs/SRs.

Characteristic	N	%
Publication year		
2012	1	5.9
2013	1	5.9
2014	1	5.9
2017	2	11.8
2019	1	5.9
2020	1	5.9
2021	1	5.9
2022	8	47.1
2024	1	5.9
Country of corresponding author		
China	14	82.4
China-Taiwan	1	5.9
Jordan	1	5.9
Netherlands	1	5.9
Subject characteristics		
Acute	0	0.0
Subacute	0	0.0
Chronic	1	5.9
Mixed	16	94.1
Not reported	0	0.0
Study design		
MAs/SRs of RCTs	16	94.1
MAs/SRs of RCTs and crossover RCTs	1	5.9
Intervention description reported		
Description reported	10	58.9
Funding		
Yes (non-industry)	10	58.9
No	1	5.9
Not reported	6	35.3
Outcomes reported		
Upper limb motor function	9	52.9
Hand dexterity	11	64.7
Hand strength	4	23.5
ADL	4	23.5
Stroke severity	2	11.8
Upper limb spasticity	6	35.3
Cortical excitability	5	29.4
Adverse events	1	58.8
Meta-analysis performed		
Yes	16	94.1
No	1	5.9

MAs/SRs, meta-analyses and systematic reviews; RCTs, Randomized Controlled Trials; ADL, Activity of Daily Living.

### 3.3 Outcome measures

**Figure 2:**
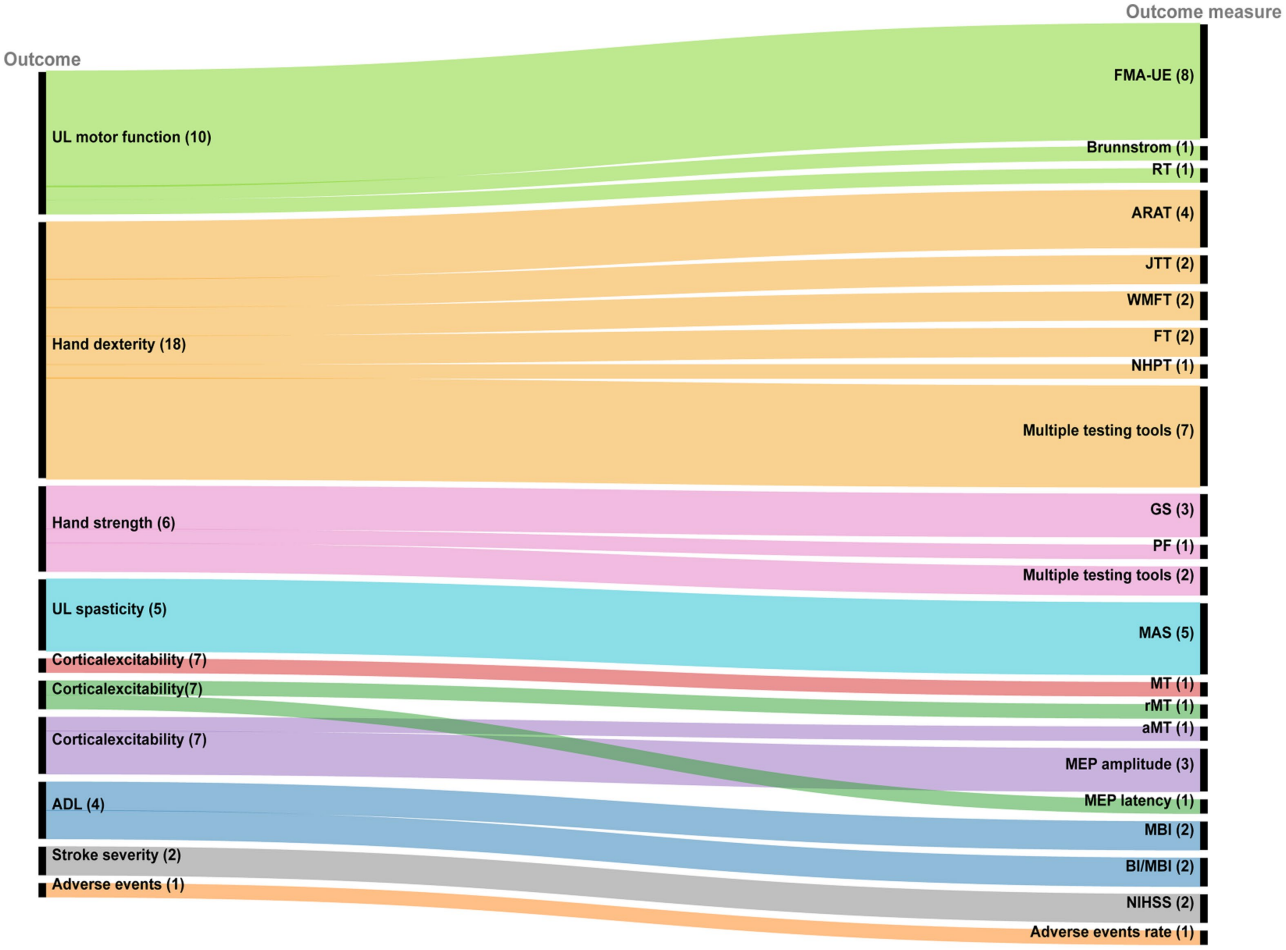
Outcome measures chart reported by the meta-analysis. FMA-UE, Fugl-Meyer Assessment of Upper Extremity; RT, Reaching Time; ARAT, Action Research Arm Test; JTT, Jebsen-Taylor Test; WMFT, Wolf Motor Function Test; FT, Finger Tapping; NHPT, Nine-Hole Peg Test; GS, Grip strength; PF, Pinch Force; MAS, Modified Ashworth; MT, Motor Threshold; rMT, resting Motor Threshold; aMT, active Motor Threshold; MEP, Motor Evoked Potential; MBI, Modified Barthel Index; BI, Barthel Index; NIHSS, National Institute of Health Stroke Scale.

The outcome measures synthesized across the included MAs/SRs are visually represented in Figure 2. This Sankey diagram illustrates the hierarchical flow from recovery domains to specific instruments, highlighting a clear predominance of motor function metrics over neurophysiological or ADL indicators. The Fugl-Meyer Assessment–Upper Extremity (FMA-UE) was the most frequently used instrument to evaluate upper limb motor function, whereas hand dexterity and hand strength were assessed using a variety of validated tools across the studies.

### 3.4 Publication year of included studies

A total of 17 MAs/SRs, spanning from 2012 to 2024, were included in this overview. The temporal distribution reveals a notable surge in research interest recently, with nearly half of the included studies (35–38, 43, 45, 49, 50) published in 2022. Two reviews (46, 48) were published in 2017, with the remaining reviews distributed across seven different years (39–42, 44, 47, 51). This distribution suggests a growing body of evidence on the application of rTMS in stroke rehabilitation over the past decade.

### 3.5 Study type of included studies

Randomized controlled trials are widely regarded as the gold standard for evaluating treatment efficacy. In this overview, most included MAs/SRs were based exclusively on RCTs (n = 16, 94.1%), whereas one review (51) included both parallel-group and crossover RCTs. This predominance of RCT-based evidence strengthens the overall evidence base for evaluating the effectiveness of rTMS interventions.

### 3.6 The AMSTAR-2 score

The AMSTAR 2 scores of the 17 included MAs/SRs ranged from 3.5 to 10.5, and all were rated as having very low methodological quality (see Table 3 for details). The critical entries reported with notable deficiencies are entry 2 (35.3%), entry 7 (0%), entry 13 (41.2%), and entry 15 (35.3%). Refer to Supplementary Table 5 for details. Several key methodological limitations contributed to these deficiencies. First, only six reviews (36, 37, 39, 41, 43, 46) had prospectively registered protocols, and none adequately explained deviations from their predefined study protocols. Second, most reviews did not report consultation with subject experts, did not justify restrictions related to search language or time limits, and rarely searched for gray literature. Third, none of the reviews provided a complete list of excluded studies with reasons for exclusion. Although most reviews applied appropriate statistical methods for meta-analysis, only eight reviews (36, 38, 40, 42, 43, 46, 48, 50) further explored the sources of heterogeneity or attempted to control for potential confounding factors. In addition, publication bias was insufficiently addressed. Ten reviews (35, 37, 40–45, 48, 49) did not discuss the potential impact of publication bias in small-sample studies, and only six reviews (39, 40, 44, 46, 50, 51) assessed publication bias using statistical tests or funnel plots. For the non-critical domains, substantial reporting deficiencies were also observed in Item 3 (0%), Item 10 (5.9%), Item 12 (29.4%), and Item 16 (35.3%). None of the reviews provided a clear rationale for the selection of study designs. Only one reviews (44) reported the funding sources of the included studies. Furthermore, only five reviews (38, 39, 48, 50, 51) assessed how the risk of bias in the included studies might have influenced the meta-analysis results. Although most reviews reported funding sources and conflicts of interest, few clearly described the role of funders in the research process.

**Table 3:** Multivariate evaluation and ranking of the six dimensions across included studies (*N* = 17).

Study ID	Publication year	Study type	AMSTAR-2 scale	PRISMA 2020 scale	Homogeneity	Publication bias	Average rank score
Le et al. (44)	2013 (2)	RCTs (17)	3.5 (1)	13 (2)	High (17)	None (11)	8.33
Ling et al. (36)	2017 (5)	RCTs (17)	9.5 (15)	14.5 (5)	High (17)	None (11)	11.67
Wu et al. (37)	2022 (16)	RCTs (17)	4.5 (2)	13.5 (3)	Low (6)	None (11)	9.17
Xia et al. (38)	2022 (16)	RCTs (17)	10.5 (17)	18 (10)	Low (6)	Funnel plot (16)	13.67
Gao (39)	2021 (8)	RCTs and crossover RCTs (1)	10 (16)	16.5 (8)	High (17)	Funnel plot (16)	11.00
Hsu et al. (40)	2012 (1)	RCTs (17)	8.5 (9)	14.5 (5)	High (17)	Egger’s test (16)	10.83
Chen et al. (41)	2022 (16)	RCTs (17)	9 (13)	18.5 (11)	Low (6)	None (11)	12.33
Tang et al. (42)	2022 (16)	RCTs (17)	7 (7)	19.5 (13)	High (17)	None (11)	13.50
Zhang et al. (43)	2017 (5)	RCTs (17)	9.5 (15)	19 (12)	High (17)	Trim and fill method (16)	13.67
Le et al. (35)	2014 (3)	RCTs (17)	5 (3)	15.5 (6)	High (17)	None (11)	9.50
Alashram et al. (45)	2022 (16)	RCTs (17)	5.5 (4)	12 (1)	None (1)	None (11)	8.33
He et al. (46)	2020 (7)	RCTs (17)	9 (13)	20.5 (15)	High (17)	Funnel plot and Egger’s test (17)	14.33
van Lieshout et al. (47)	2019 (6)	RCTs (17)	7.5 (8)	20 (14)	High (17)	Funnel plot (16)	13.00
Chen et al. (48)	2022 (16)	RCTs (17)	6.5 (6)	21 (16)	Low (6)	None (11)	12.00
Jiang et al. (49)	2024 (17)	RCTs (17)	9 (13)	21.5 (17)	High (17)	None (11)	15.33
Wang et al. (50)	2022 (16)	RCTs (17)	6 (5)	17 (9)	High (17)	None (11)	12.50
Gao et al. (51)	2022 (16)	RCTs (17)	9 (13)	16.5 (8)	Low (6)	None (11)	11.83
Average rank score of evaluation dimensions	10.71	16.06	9.41	9.12	12.82	12.82	11.82

RCTs, Randomized Controlled Trials; None, indicates that publication bias assessment was not stated or performed. Numbers in parentheses represent the rank of each study across all 17 included reviews (1 = lowest, 17 = highest).

### 3.7 The PRISMA 2020 score

The PRISMA reporting scores of the 17 included MAs/SRs ranged from 12 to 21.5, indicating suboptimal reporting quality overall, Refer to Table 3 for details. The items most frequently underreported were Item 7 (search strategy, 41.2%), Item 27 (availability of data or materials, 47.1%), Item 21 (risk of bias across studies, 35.3%), and Item 25 (funding, 32.4%), followed by Item 15 (certainty of evidence, 17.6%), Item 22 (grading of evidence quality, 17.6%), and Item 24 (additional information, 17.6%), Refer to Supplementary Table 6 for details. Specific methodological deficits included: First, except for four reviews (36, 37, 43, 46), most abstracts lacked sufficient detail regarding study objectives, methods, and key findings. Second, 11 reviews (35, 38, 40, 42, 44, 45, 47–51) did not provide protocol registration numbers. Third, detailed descriptions of the literature search process were frequently missing, including databases searched, search dates, and reference list screening. In addition, eight reviews (35, 38, 40, 42, 45–48) did not report complete search strategies for all databases or platforms, including applied filters or search limits. Definitions of outcome measures and assumptions regarding missing or unclear data were also rarely reported, with only one review (44) providing detailed descriptions. Furthermore, only three reviews (36, 39, 41) described methods used to assess the certainty of outcome evidence. Publication bias was insufficiently evaluated, as only six reviews (39, 40, 44, 46, 50, 51) conducted statistical tests or used funnel plots to assess potential bias. Funding transparency was also limited; six reviews (35, 39, 42, 47, 48, 51) did not disclose funding sources, and those that did rarely described the role of funders. Finally, nine reviews (35, 40, 42, 45, 47–51) did not report the availability of publicly accessible data or supporting materials.

### 3.8 Homogeneity

Among the 17 included reviews, in 11 studies (39–48, 51), low statistical heterogeneity (*p* ≥ 0.10 and I^2^ ≤ 50%) was observed in more than half of the reported outcome measures, indicating relatively consistent results across the included primary studies. Five reviews (36–38, 49, 50) reported substantial heterogeneity in more than half of their outcome measures. One review (35) did not perform a meta-analysis. Detailed information is presented in Table 3.

### 3.9 The publication bias reports

Publication bias was assessed in only a small proportion of the included reviews. One review (39) evaluated publication bias using both funnel plots and Egger’s test. Another review (46) applied the trim-and-fill method, while one review (40) used Egger’s test alone. Three review (44, 50, 51) assessed publication bias using funnel plots. The remaining reviews did not report an assessment of publication bias. Detailed information is provided in Table 3.

### 3.10 Multidimensional assessment using radar plots

#### 3.10.1 Visual interpretation of radar plots

**Figure 3:**
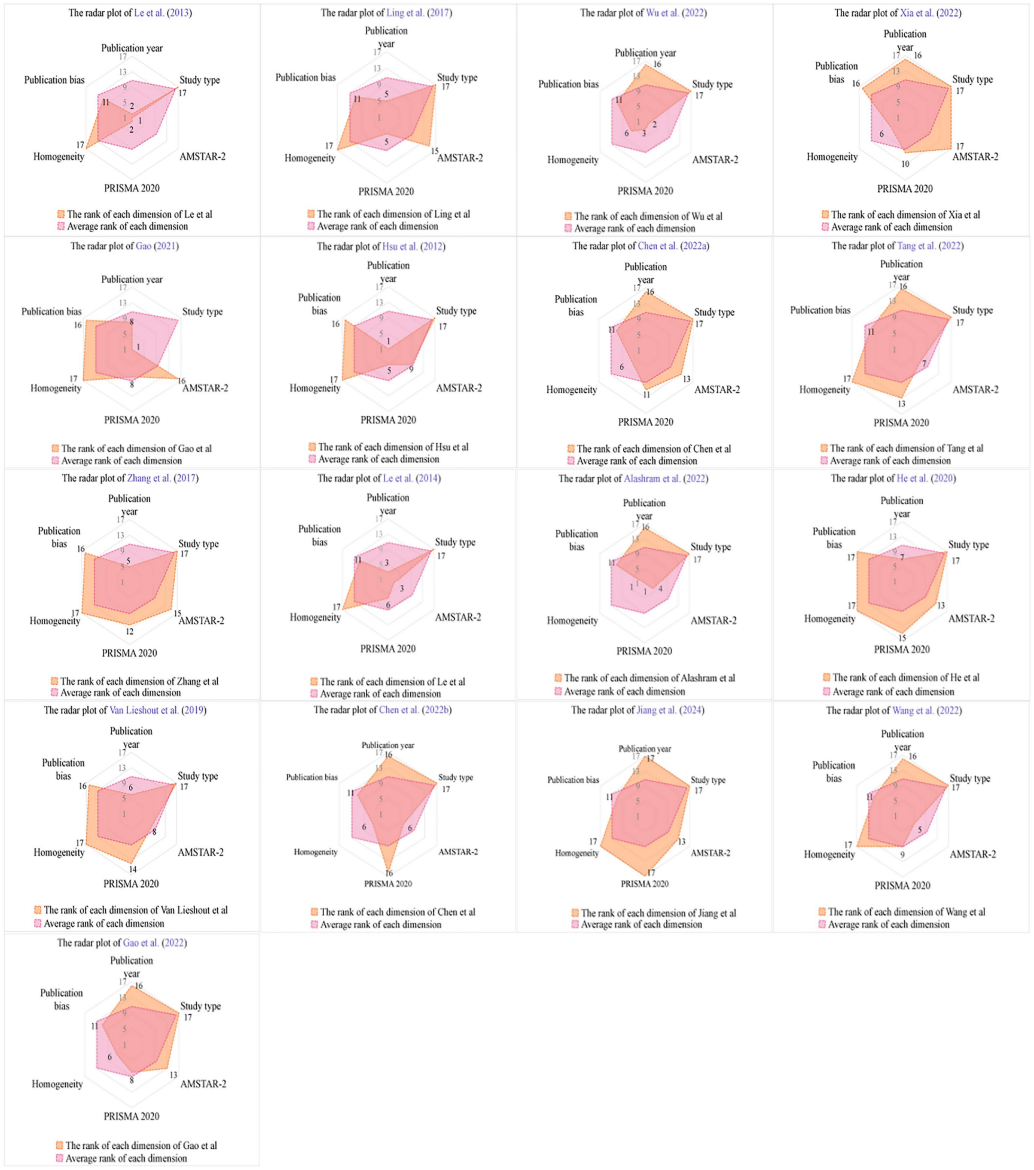
Orange shading represents the rank of each evaluation dimension for each included review, whereas pink shading indicates the mean rank across all included reviews.

Table 3 presents the ranking results across the six evaluation dimensions. A higher mean rank indicates better overall quality. Radar plots were constructed based on these rankings, with a larger enclosed area representing higher methodological and reporting quality. Visual inspection of the radar plots indicates that none of the included MAs/SRs achieved above-average rankings across all six dimensions, suggesting that each review had methodological or reporting limitations affecting its overall quality. Three reviews (41, 46, 50) performed relatively well, ranking above the average in five of the six dimensions and demonstrating comparatively large radar plot areas. These reviews may therefore provide relatively stronger evidence for reference. Another three reviews (37, 43, 44) ranked above the average in four dimensions and showed moderately large radar plot areas, indicating intermediate overall quality. In contrast, the remaining reviews ranked below the average in more than half of the evaluated dimensions and displayed comparatively small radar plot areas, suggesting relatively lower overall quality. Consequently, the findings of these reviews should be interpreted with caution. Detailed radar plot results are presented in Figure 3. Overall, the methodological and reporting quality of MAs/SRs published between 2012 and 2024 shows considerable variability, indicating substantial room for improvement in future evidence syntheses.

### 3.11 GARDE evidence quality grading

A total of 222 outcomes were extracted from the 17 included MAs/SRs. The certainty of evidence for each outcome was assessed using the GRADE approach. Among these outcomes, 13 were rated as high quality evidence, 47 as moderate quality evidence, 91 as low quality evidence, and 71 as very low quality evidence, indicating that the overall certainty of evidence remains limited. Detailed results are presented in Supplementary Table 7.

## 4 Safety and adverse events

Nine reviews (35, 37–40, 42, 43, 47, 48) documented the safety profiles associated with rTMS interventions. Commonly reported adverse effects were predominantly mild and transient, including scalp tingling, tension-type headaches, dizziness, and localized discomfort (e.g., non-specific neck pain). These symptoms typically resolved spontaneously following a brief rest period, requiring no specialized medical intervention. Regarding serious adverse events (SAEs), two reviews (35, 43) identified rare instances of induced seizures or epileptiform activity. However, such occurrences remained exceptional within the broader evidence base. Collectively, the synthesized data underscore that rTMS is a well-tolerated and clinically safe modality for post-stroke upper limb rehabilitation when administered within established safety parameters.

## 5 Discussion

### 5.1 Summary of evidence

This study reviewed 17 MAs/SRs published between 2012 and 2024 (35–51) including five (47–51) in Chinese and 12 (35–46) in English. In terms of study design, most studies primarily focused on RCTs, with only one study (51) incorporating both RCTs and crossover RCTs. The average rank scores across the six evaluated dimensions ranged from 8.33 to 15.33, with an overall mean of 11.82; Jiang et al. (49) achieved the highest quality score, while Le et al. (44) and Alashram et al. (45) were rated lowest.

Methodological quality, assessed using AMSTAR-2, ranged from 3.5 to 10.5, and reporting quality, assessed via PRISMA 2020, ranged from 12 to 21.5. Most studies lacked subgroup, regression, or sensitivity analyses to explore the influence of bias on overall effect estimates. Heterogeneity was reported in 10 (36–41, 45, 48, 50, 51) studies and was generally high. Only study (44) disclosed funding sources for the included studies. The primary contributors to poor quality were low methodological rigor and insufficient reporting; secondary factors included publication year and potential publication bias. Notably, all studies were rated “very low” on the AMSTAR-2 scale due to multiple unmet critical criteria.

Based on these findings, future MAs/SRs in this field should focus on the following methodological improvements: (i) Strict adherence to the PICOS framework, with clear justification for study inclusion criteria. (ii) Comprehensive literature searches, including gray literature, with explicit reporting of search strategies and restrictions. (iii) Transparent listing of included and excluded studies with reasons for exclusion. (iv) Independent study selection and data extraction by at least two reviewers. (v) Detailed characterization of included studies. (vi) Systematic assessment of risk of bias and its potential impact on meta-analytic conclusions. (vii) Clear reporting of funding sources and conflicts of interest. Additionally, improvements in reporting should include pre-registration in PROSPERO, detailed documentation of search strategies in primary databases, stepwise description of study selection, use of predefined analysis methods, disclosure of funder roles, and a comprehensive evidence summary.

### 5.2 Potential mechanisms and individual differences of rTMS in motor function recovery

Repetitive transcranial magnetic stimulation has emerged as a pivotal intervention in neurorehabilitation. While the clinical efficacy of rTMS in facilitating post-stroke motor recovery is increasingly recognized, the precise neurobiological underpinnings require further elucidation. Although interhemispheric inhibition and neural plasticity are well-established foundational mechanisms, this overview shifts the focus toward the utility of magnetic resonance imaging (MRI) biomarkers. Given the pronounced inter-individual variability in rTMS responsiveness, leveraging MRI biomarkers offers a critical pathway to decode patient-specific neurophysiological responses. Ultimately, this paradigm shift has the potential to guide personalized neuromodulatory strategies, refine clinical decision-making, and optimize healthcare resource allocation.

#### 5.2.1 Biomarkers based on diffusion tensor imaging reveal potential mechanisms of rTMS-induced motor recovery

Stroke pathophysiology extends beyond localized tissue infarction, profoundly disrupting the structural and functional integrity of widespread neural networks. In this context, advanced non-invasive neuroimaging techniques offer quantifiable windows into post-stroke neuroplasticity. Specifically, diffusion tensor imaging (DTI) provides critical metrics of white matter microstructural integrity—such as fractional anisotropy (FA) and mean diffusivity (MD)—while functional MRI (fMRI) maps temporal changes in cortical activation and functional connectivity. By serving as objective neuroimaging biomarkers, these DTI and fMRI indices are indispensable for decoding the neural mechanisms by which rTMS drives motor recovery, facilitating the transition from clinical observation to precision neuromodulation (52, 53).

Emerging evidence from diffusion tensor imaging (DTI) paradigms underscores the capacity of rTMS to drive structural neuroplasticity. Specifically, Yamada et al. (54) reported that low-frequency rTMS applied to the unaffected hemisphere increased fractional anisotropy (FA) and generalized FA (GFA) in the bilateral Brodmann area 4 (BA4), with GFA alterations in the affected hemisphere directly associated with motor recovery. Complementing this, high-frequency rTMS targeting the affected primary motor cortex (M1) has been shown to significantly enhance Fugl-Meyer Assessment (FMA) scores compared to conventional therapy (55). Crucially, this clinical improvement significantly correlated with pronounced FA increases in the affected posterior limb of the internal capsule. Furthermore, Li et al. (56) observed that such high-frequency stimulation also elevated FA values across widespread networks, including the pons-cerebellum-cortical loop, corpus callosum, and cingulate gyrus. Collectively, these neuroimaging findings provide robust mechanistic evidence that rTMS facilitates post-stroke motor recovery by inducing structural reorganization within the corticospinal tract and enhancing white matter connectivity across critical motor networks, including bilateral M1 and cortico-cerebellar pathways.

#### 5.2.2 Biomarkers based on functional magnetic resonance imaging reveal mechanisms of rTMS-induced motor recovery

Functional magnetic resonance imaging studies suggest that rTMS effectively modulates cortical activation and functional connectivity (FC) within motor-related networks following stroke. Investigating this bimodal neuromodulation, Du et al. (57) and Bao et al. (58) demonstrated that low-frequency rTMS applied to the unaffected M1 and high-frequency rTMS applied to the affected M1 elicit distinct, yet complementary, activation patterns. Specifically, high-frequency stimulation upregulated activation in the affected M1, whereas low-frequency stimulation significantly suppressed hyperactivation in the unaffected M1. Importantly, this rebalancing of M1 excitability was significantly correlated with enhanced motor performance. Building upon these focal effects, Ueda et al. (59) observed that low-frequency rTMS to the unaffected M1 significantly strengthened FC between bilateral M1 regions. Similarly, in a cohort with acute subcortical stroke, Guo et al. (55) reported that both high- and low-frequency rTMS protocols yielded superior motor recovery compared to sham interventions. This clinical improvement was underpinned by enhanced FC across the broader motor network, encompassing bilateral M1, the SMA, and the premotor cortex (PMC). Notably, high-frequency rTMS induced a more pronounced increase in FC between the affected M1 and the contralateral PMC, a neuroplastic shift directly associated with motor gains. Collectively, these neuroimaging findings elucidate a crucial neural mechanism: rTMS drives motor recovery not merely by inducing focal cortical excitation, but by facilitating robust functional reorganization and network-level rebalancing across bilateral motor circuits.

#### 5.2.3 MRI-based biomarkers reveal individual differences in rTMS treatment response

Although numerous studies have demonstrated that rTMS can facilitate motor recovery after stroke, several challenges remain in clinical application, including identifying suitable candidates for treatment, selecting personalized stimulation protocols, and predicting therapeutic responses. Neuroimaging biomarkers derived from magnetic resonance imaging may help address these challenges. Diekhoff-Krebs et al. (60) reported that stroke patients with better hand motor function exhibited stronger functional connectivity between the SMA and the M1 of the affected hemisphere prior to rTMS treatment. Importantly, post-treatment behavioral improvements were associated with the strength of pre-treatment neural coupling within the stimulated hemisphere, particularly connections targeting the affected M1. By integrating intrinsic connectivity measures with behavioral parameters, the authors were able to explain approximately 82% of the variability in hand motor performance following rTMS in patients with hemiparetic stroke. Additional evidence highlighting the role of individual differences comes from the work of Quinlan et al. (61). In their study of upper limb robot-assisted therapy, stroke patients with severe motor impairment (FMA-UE ≤ 36) showed greater motor improvement when functional connectivity increased, whereas patients with milder impairment (FMA-UE > 36) exhibited greater motor gains when functional connectivity decreased. Although this study did not directly examine rTMS, the findings support the notion that the contribution of the contralesional hemisphere to motor recovery varies depending on impairment severity. These findings have important implications for rTMS treatment strategies. In patients with mild motor impairment, inhibitory rTMS applied to the unaffected hemisphere may help restore interhemispheric balance and optimize motor recovery. In contrast, in patients with severe impairment, the unaffected hemisphere may play a compensatory role in supporting motor function. In such cases, suppressing activity in the unaffected hemisphere may be counterproductive, and therapeutic strategies that enhance excitability in the affected hemisphere may be more appropriate. Overall, MRI-based biomarkers provide valuable neurobiological insights into individual variability in behavioral responses to rTMS and may facilitate the development of personalized neuromodulation strategies for stroke rehabilitation.

In summary, MRI-derived biomarkers offer profound neurobiological insights into the mechanisms of rTMS-induced motor recovery and provide a critical framework for decoding inter-individual variability in treatment efficacy. Despite this potential, the current evidence base lacks stage-specific granular data across the temporal continuum of stroke recovery. Moving forward, the paradigm must shift toward Precision Neurorehabilitation. Future research should prioritize the validation of imaging biomarkers that can effectively stratify patients based on residual structural integrity and baseline functional connectivity. Such a biomarker-guided approach will be instrumental in identifying optimal responders, refining stimulation targets, and monitoring the long-term durability of therapeutic gains. Ultimately, integrating neuroimaging-guided strategies into clinical decision-making will facilitate the transition from empirical rTMS application to personalized neuromodulatory prescriptions, thereby maximizing the functional independence of stroke survivors.

### 5.3 Applicability and implications for future research

The aggregated findings from the included MAs/SRs unequivocally support the efficacy of rTMS in driving upper-limb motor recovery. However, the therapeutic effect size is highly contingent upon a complex interplay of clinical moderators:

(1) Temporal dynamics and the “recovery window.” The evidence elucidates a distinct time-dependent efficacy gradient: neuroplastic responsiveness is most robust during the acute phase (< 1 month), tapers through the subacute stage, and reaches a functional plateau in chronic stroke (> 6 months) (40, 44). Critically, excitatory protocols appear to have a “priming window” limited to the first 90 days post-insult (43). Stimulation strategies must therefore be phase-specific: (i) Acute Phase: Favors high-intensity, bilateral recruitment (≥ 20 sessions). (ii) Subacute Phase: Shifts toward sustained ipsilesional reinforcement (up to 40 sessions). (iii) Chronic Phase: Prioritizes contralesional inhibition (e.g., 10 sessions) to refine interhemispheric balance (36).
(2) Paradigm optimization: frequency and waveforms. Regarding frequency, low-frequency rTMS (LF-rTMS) exhibits a superior clinical edge (47, 48), effectively decoupling the over-active unaffected hemisphere without compromising its inherent dexterity (40). While iTBS generally demonstrates higher potency than continuous TBS (41, 50), its application is stage-dependent. TBS, characterized by rapid plasticity induction, is the optimal modality for the acute window, whereas conventional rTMS protocols offer more consistent benefits for patients in more stable, chronic stages (36).
(3) Dosage and lesion-specific responsiveness. A dosage of 600 pulses represents a “optimal therapeutic threshold,” yielding maximal functional increments while avoiding the inhibitory fatigue associated with excessive pulse counts (37, 38). Furthermore, lesion topography significantly influences outcomes: patients with subcortical involvement exhibit higher responsiveness compared to those with non-specific cortical lesions (39). Encouragingly, these therapeutic gains remain resilient across stroke etiologies, showing consistent efficacy in both ischemic and hemorrhagic subtypes (51).
(4) Management of spasticity: the multi-modal necessity. The evidence for spasticity modulation remains convoluted. While multimodal integration (rTMS coupled with conventional rehabilitation) is significantly superior to monotherapy (35, 45)—even into the chronic phase (37)—the role of rTMS or iTBS as a standalone intervention remains contentious. Several high-quality analyses report negligible benefits, suggesting that spasticity relief requires a synergistic “top-down” (TMS) and “bottom-up” (rehabilitation) approach (38, 49).

## 6 Limitations

The findings of this overview should be interpreted within the context of several inherent limitations. First, the search was confined to studies published in English and Chinese. While this addressed feasibility, the exclusion of other languages may have introduced language and selection bias, potentially omitting relevant evidence from other geographic regions. Second, as is common in overviews, there was a potential overlap of primary studies across the included meta-analyses. The recurrent synthesis of identical randomized controlled trials (RCTs) across multiple reviews may artificially inflate the perceived volume of evidence and diminish the independence of pooled estimates. Notably, a formal quantitative assessment of overlap (e.g., calculation of the Corrected Covered Area) was precluded by the incomplete primary study lists in several reviews. Third, significant clinical heterogeneity was observed in rTMS protocols across the analyzed literature. Substantial variability in key stimulation parameters—including frequency, intensity, pulse numbers, and anatomical targets—complicates cross-study comparability and limits the formulation of standardized clinical pathways. Such heterogeneity was a primary driver for downgrading evidence certainty in the GRADE assessments. Fourth, the methodological quality of the included reviews was variable; particularly, the suboptimal reporting of funding sources and conflicts of interest may affect the transparency of the synthesized findings. Finally, while adverse events were reported, the documentation was often inconsistent and lacked standardized definitions, severity grading, or quantitative incidence data. This lack of systematic monitoring precluded a formal meta-analysis of safety outcomes, thereby limiting a comprehensive evaluation of the risk–benefit profile of rTMS in stroke rehabilitation.

## 7 Conclusion

In summary, while current MAs/SRs provide encouraging evidence that rTMS significantly enhances upper limb motor recovery post-stroke, the clinical translation of these findings is currently hampered by modest methodological rigor and inconsistent reporting standards. To bridge the gap between clinical research and evidence-based practice, it is imperative that future investigators strictly adhere to AMSTAR-2, PRISMA 2020, and GRADE frameworks from the protocol inception phase. Furthermore, we advocate for a structural shift in the academic publishing ecosystem: journal editors should mandate the use of standardized reporting checklists to ensure transparency and reproducibility. Such concerted efforts across the research community are essential to refine the certainty of evidence, ultimately facilitating the evolution from generalized stimulation protocols to biomarker-driven, personalized neuromodulation strategies in stroke rehabilitation. The current absence of objective MRI-derived biomarkers, such as functional connectivity (SMA-M1) or structural integrity (Corticospinal Tract), likely contributes to the high inter-study heterogeneity and subsequent downgrading of evidence certainty in GRADE assessments.

## Data Availability

The original contributions presented in the study are included in the article/Supplementary material, further inquiries can be directed to the corresponding author.

## Footnotes

1
www.opengrey.eu
