# Supplementary material for: Effects of repetitive transcranial magnetic stimulation on upper limb motor recovery after stroke: an overview of systematic reviews
Source: Front Neurol. 2026 Jun 10;17:1797218. doi: 10.3389/fneur.2026.1797218 (PMC13292297; doi:10.3389/fneur.2026.1797218)
Supplement: Supplementary file 1 [file Table_1.docx]

Supplementary Material

# 1　　Supplementary Tables

**Supplementary Table S1.　Search strategy (****Pubmed database retrieval strategies).**

| **Database** | **Retrieval strategy** | **Date of search** |
| --- | --- | --- |
| PubMed | #1 "Stroke"[Mesh]  #2 Strokes[Title/Abstract] OR Cerebrovascular Accident[Title/Abstract] OR Cerebrovascular Accidents[Title/Abstract] OR CVA (Cerebrovascular Accident[Title/Abstract]) OR CVAs (Cerebrovascular Accident[Title/Abstract]) OR Cerebrovascular Apoplexy[Title/Abstract] OR Apoplexy, Cerebrovascular[Title/Abstract] OR Vascular Accident, Brain[Title/Abstract] OR Brain Vascular Accident[Title/Abstract] OR Brain Vascular Accidents[Title/Abstract] OR Vascular Accidents, Brain[Title/Abstract] OR Cerebrovascular Stroke[Title/Abstract] OR Cerebrovascular Strokes[Title/Abstract] OR Stroke, Cerebrovascular[Title/Abstract] OR Strokes, Cerebrovascular[Title/Abstract] OR Apoplexy[Title/Abstract] OR Cerebral Stroke[Title/Abstract] OR Cerebral Strokes[Title/Abstract] OR Stroke, Cerebral[Title/Abstract] OR Strokes, Cerebral[Title/Abstract] OR Stroke, Acute[Title/Abstract] OR Acute Stroke[Title/Abstract] OR Acute Strokes[Title/Abstract] OR Strokes, Acute[Title/Abstract] OR Cerebrovascular Accident, Acute[Title/Abstract] OR Acute Cerebrovascular Accident[Title/Abstract] OR Acute Cerebrovascular Accidents[Title/Abstract] OR Cerebrovascular Accidents, Acute[Title/Abstract]  #3 #1 OR #2  #4 "Transcranial Magnetic Stimulation"[Mesh]  #5 Magnetic Stimulation, Transcranial[Title/Abstract] OR Magnetic Stimulations, Transcranial[Title/Abstract] OR Stimulation, Transcranial Magnetic[Title/Abstract] OR Stimulations, Transcranial Magnetic[Title/Abstract] OR Transcranial Magnetic Stimulations[Title/Abstract] OR Transcranial Magnetic Stimulation, Single Pulse[Title/Abstract] OR Transcranial Magnetic Stimulation, Paired Pulse[Title/Abstract] OR Transcranial Magnetic Stimulation, Repetitive[Title/Abstract] OR repetitive transcranial magnetic stimulation[Title/Abstract] OR TMS[Title/Abstract] OR rTMS[Title/Abstract] OR magnetic stimulation[Title/Abstract] OR TBS[Title/Abstract] OR iTBS[Title/Abstract] OR cTBS[Title/Abstract] OR Theta burst stimulation[Title/Abstract] OR intermittent theta burst stimulation[Title/Abstract] OR continuous theta burst stimulation[Title/Abstract] OR non-invasive brain stimulation[Title/Abstract]  #6 #4 OR #5  #7 "Upper Extremity"[Mesh]  #8 Manual dexterity[Title/Abstract] OR motor movement[Title/Abstract] OR hand dexterity[Title/Abstract] OR motor skills[Title/Abstract] OR Upper limb[Title/Abstract] OR upper extremity[Title/Abstract]  #9 #7 OR #8  #10 "Hand"[Mesh]  #11 hand[Title/Abstract] OR hands[Title/Abstract] OR arm[Title/Abstract]  #12 #10 OR #11  #13 #9 OR #12  #14 "Systematic Review"[Publication Type]  #15 "Meta-Analysis"[Publication Type]  #16 meta-analysis[Title/Abstract] OR meta analysis[Title/Abstract] OR systematic review[Title/Abstract] OR review Overview, Clinical Trial[Title/Abstract] OR Clinical Trial Overview[Title/Abstract]  #17 #14 OR #15 OR #16  #18 #3 AND #6 AND #13 AND #17 | April 1, 2024 |

**Supplementary Table S2.　Search strategy (common database retrieval strategies).**

| **Database** | **Retrieval strategy** | **Date of search** |
| --- | --- | --- |
| Embase | #1 'cerebrovascular accident'/exp  #2 'strokes':ab,ti OR 'cerebrovascular accident':ab,ti OR 'cerebrovascular accidents':ab,ti OR 'cva (cerebrovascular accident)':ab,ti OR 'cvas (cerebrovascular accident)':ab,ti OR 'cerebrovascular apoplexy':ab,ti OR 'apoplexy, cerebrovascular':ab,ti OR 'vascular accident, brain':ab,ti OR 'brain vascular accident':ab,ti OR 'brain vascular accidents':ab,ti OR 'vascular accidents, brain':ab,ti OR 'cerebrovascular stroke':ab,ti OR 'cerebrovascular strokes':ab,ti OR 'stroke,cerebrovascular':ab,ti OR 'strokes, cerebrovascular':ab,ti OR 'apoplexy':ab,ti OR 'cerebral stroke':ab,ti OR 'cerebral strokes':ab,ti OR 'stroke, cerebral':ab,ti OR 'strokes, cerebral':ab,ti OR 'stroke, acute':ab,ti OR 'acute stroke':ab,ti OR 'acute strokes':ab,ti OR 'strokes, acute':ab,ti OR 'cerebrovascular accident, acute':ab,ti OR 'acute cerebrovascular accident':ab,ti OR 'acute cerebrovascular accidents':ab,ti OR 'cerebrovascular':ab,ti  #3 'transcranial magnetic stimulation'/exp  #4 'magnetic stimulation, transcranial':ab,ti OR 'magnetic stimulations, transcranial':ab,ti OR 'stimulation, transcranial magnetic':ab,ti OR 'stimulations, transcranial magnetic':ab,ti OR 'transcranial magnetic stimulations':ab,ti OR 'transcranial magnetic stiulatimon, single pulse':ab,ti OR 'transcranial magnetic stimulation, paired pulse':ab,ti OR 'transcranial magnetic stimulation, repetitive':ab,ti OR 'tms':ab,ti OR 'rtms':ab,ti OR 'magnetic stimulation':ab,ti OR 'tbs':ab,ti OR 'itbs':ab,ti OR 'ctbs':ab,ti OR 'theta burst stimulation':ab,ti OR 'intermittent theta burst stimulation':ab,ti OR 'continuous theta burst stimulation':ab,ti OR 'non-invasive brain stimulation':ab,ti  #5 'upper extremity'/exp  #6 'manual dexterity':ab,ti OR 'motor movement':ab,ti OR 'hand dexterity':ab,ti OR 'motor skills':ab,ti OR 'upper limb':ab,ti OR 'upper extremity':ab,ti  #7 'hand'/exp  #8 'hand':ab,ti OR 'hands':ab,ti OR 'arm':ab,ti  #9 'systematic review'/exp  #10 'meta analysis'/exp  #11 'meta-analysis':ab,ti OR 'meta analysis':ab,ti OR 'systematic review':ab,ti OR 'review overview, clinical trial':ab,ti OR 'clinical trial overview':ab,ti  #12 #1 OR #2  #13 #3 OR #4  #14 #5 OR #6  #15 #7 OR #8  #16 #14 R #15  #17 #9 OR #10 OR #11  #18 #12 AND #13 AND #16 AND #17 | April 1, 2024 |
| Web of Science | #1 TS =Strokes or Cerebrovascular Accident or Cerebrovascular Accidents or CVA (Cerebrovascular Accident) or CVAs (Cerebrovascular Accident) or Cerebrovascular Apoplexy or Apoplexy, Cerebrovascular or Vascular Accident, Brain or Brain Vascular Accident or Brain Vascular Accidents or Vascular Accidents, Brain or Cerebrovascular Stroke or Cerebrovascular Strokes or Stroke, Cerebrovascular or Strokes, Cerebrovascular or Apoplexy or Cerebral Stroke or Cerebral Strokes or Stroke, Cerebral or Strokes, Cerebral or Stroke, Acute or Acute Stroke or Acute Strokes or Strokes, Acute or Cerebrovascular Accident, Acute or Acute Cerebrovascular Accident or Acute Cerebrovascular Accidents or Cerebrovascular Accidents, Acute  #2 TS =transcranial magnetic stimulation or Magnetic Stimulation, Transcranial or Magnetic Stimulations, Transcranial or Stimulation, Transcranial Magnetic or Stimulations, Transcranial Magnetic or Transcranial Magnetic Stimulations or Transcranial Magnetic Stimulation, Single Pulse or Transcranial Magnetic Stimulation, Paired Pulse or Transcranial Magnetic Stimulation, Repetitive or TMS or rTMS or magnetic stimulation or TBS or iTBS or cTBS or theta burst stimulation or intermittent theta burst stimulation or continuous theta burst stimulation or non-invasive brain stimulation  #3 TS =manual dexterity OR motor movement OR hand dexterity OR motor skills OR upper limb OR upper extremity OR upper extremity OR hand OR hand OR hands  #4 TS =meta-analysis or meta analysis or systematic review or review Overview, Clinical Trial or Clinical Trial Overview  #1 AND #2 AND #3 AND #4 |  |
| Cochrane Database  of Systematic Reviews | #1 MeSH descriptor: [stroke] explode all trees  #2 (Strokes):ab,ti,kw or (Cerebrovascular Accident):ab,ti,kw or (Cerebrovascular Accidents):ab,ti,kw or (CVA (Cerebrovascular Accident)):ab,ti,kw or (CVAs (Cerebrovascular Accident)):ab,ti,kw or (Cerebrovascular Apoplexy):ab,ti,kw or (Apoplexy, Cerebrovascular):ab,ti,kw or (Vascular Accident, Brain):ab,ti,kw or (Brain Vascular Accident):ab,ti,kw or (Brain Vascular Accidents):ab,ti,kw or (Vascular Accidents, Brain):ab,ti,kw or (Cerebrovascular Stroke):ab,ti,kw or (Cerebrovascular Strokes):ab,ti,kw or (Stroke, Cerebrovascular):ab,ti,kw or (Strokes, Cerebrovascular):ab,ti,kw or (Apoplexy):ab,ti,kw or (Cerebral Stroke):ab,ti,kw or (Cerebral Strokes):ab,ti,kw or (Stroke, Cerebral):ab,ti,kw or (Strokes, Cerebral):ab,ti,kw or (Stroke, Acute):ab,ti,kw or ( Acute Stroke):ab,ti,kw or (Acute Strokes):ab,ti,kw or (Strokes, Acute):ab,ti,kw or (Cerebrovascular Accident, Acute):ab,ti,kw or (Acute Cerebrovascular Accident):ab,ti,kw or (Acute Cerebrovascular Accidents):ab,ti,kw or ( Cerebrovascular Accidents, Acute):ab,ti,kw (cognitive dysfunction):ab,ti,kw OR (cognitive impairments):ab,ti,kw  #3 #1 OR #2  #4 MeSH descriptor: [Transcranial Magnetic Stimulation] explode all trees  #5 (Magnetic Stimulation, Transcranial):ab,ti,kw or (Magnetic Stimulations, Transcranial):ab,ti,kw or (Stimulation, Transcranial Magnetic):ab,ti,kw or (Stimulations, Transcranial Magnetic):ab,ti,kw or (Transcranial Magnetic Stimulations):ab,ti,kw or (Transcranial Magnetic Stimulation, Single Pulse):ab,ti,kw or (Transcranial Magnetic Stimulation, Paired Pulse):ab,ti,kw or (Transcranial Magnetic Stimulation, Repetitive):ab,ti,kw or (repetitive transcranial magnetic stimulation):ab,ti,kw or (TMS):ab,ti,kw or (rTMS):ab,ti,kw or (magnetic stimulation):ab,ti,kw or (TBS):ab,ti,kw or (iTBS):ab,ti,kw or (cTBS):ab,ti,kw or (Theta burst stimulation):ab,ti,kw or (intermittent theta burst stimulation):ab,ti,kw or (continuous theta burst stimulation):ab,ti,kw or (non-invasive brain stimulation) ab,ti,kw  #6 #4 or #5  #7 MeSH descriptor: [Upper Extremity] explode all trees  #8 (Manual dexterity):ti,ab,kw or (motor movement):ti,ab,kw or (hand dexterity):ti,ab,kw or (motor skills):ti,ab,kw or (Upper limb):ti,ab,kw or (upper extremity):ti,ab,kw  #9 #7 or #8  #10 MeSH descriptor: [Hand] explode all trees  #11 (hand):ti,ab,kw or (hands):ti,ab,kw or (arm):ti,ab,kw  #12 #10 or #11  #13 #9 or #12  #14 MeSH descriptor: [Systematic Review] explode all trees  #15 MeSH descriptor: [Meta-Analysis]] explode all trees  #16 (meta-analysis):ab,ti,kw or (meta analysis):ab,ti,kw or (systematic review):ab,ti,kw or (review Overview, Clinical Trial):ab,ti,kw or (Clinical Trial Overview):ab,ti,kw  #17 #14 or #15 or #16  #18 #3 AND #6 AND #13 AND #17 | April 1, 2024 |
| 中国知网(CNKI） | （主题：脑卒中 + 脑出血 + 脑梗塞 + 卒中 + 中风 + 脑血管意外）AND（主题：经颅磁刺激 + rTMS + TMS + TBS + iTBS + cTBS + 重复经颅磁刺激 + 磁刺激 + 连续性θ节律性磁刺激 + 间歇性θ节律刺激 + θ刺激 + theta节律刺激 + 非入侵性脑刺激）AND（主题：上肢 + 手 + 上肢运动功能 + 运动 + 手灵活性 + 痉挛）AND（主题：系统评价 + meta分析 + 荟萃分析 + 元分析） | April 1, 2024 |
| 万方 | 检索表达式（中英文扩展&主题词扩展）：主题:(脑卒中 OR 脑出血 OR 脑梗塞 OR 脑血管意外 OR 卒中 OR 中风)*主题:(经颅磁刺激 OR rTMS OR TMS OR TBS OR iTBS OR cTBS OR 重复经颅磁刺激 OR 磁刺激 OR连续性θ节律性磁刺激 OR 间歇性θ节律刺激 OR θ刺激 OR theta节律刺激 OR 非入侵性脑刺激)*主题:(上肢 OR 手 OR 上肢运动功能 OR 运动 OR 手灵活性 OR 痉挛)*主题:(系统评价 OR meta分析 OR 荟萃分析 OR 元分析) | April 1, 2024 |
| 中国生物医学文献数据库(CBM) | #1 "卒中"[不加权:扩展]  #2 "脑卒中"[常用字段:智能] OR "脑出血"[常用字段:智能] OR "脑血管意外"[常用字段:智能] OR "卒中"[常用字段:智能] OR "中风"[常用字段:智能] OR "脑梗塞"[常用字段:智能  #3 "经颅磁刺激"[不加权:扩展]  #4 ("经颅磁刺激"[不加权:扩展]) OR ("经颅磁刺激"[常用字段:智能] OR "重复经颅磁刺激"[常用字段:智能] OR "磁刺激"[常用字段:智能] OR "连续性θ节律性磁刺激"[常用字段:智能] OR "间歇性θ节律刺激"[常用字段:智能] OR "θ刺激"[常用字段:智能] OR "theta节律刺激"[常用字段:智能] OR "非入侵性脑刺激"[常用字段:智能] OR "rTMS"[常用字段:智能] OR "TMS"[常用字段:智能] OR "TBS"[常用字段:智能] OR "iTBS"[常用字段:智能] OR "cTBS"[常用字段:智能])  #5 "上肢"[不加权:扩展]  #6 ("上肢"[常用字段:智能] OR "上肢运动功能"[常用字段:智能] OR "手"[常用字段:智能] OR "运动"[常用字段:智能] OR "手灵活性"[常用字段:智能] OR "痉挛"[常用字段:智能]) OR ("上肢"[不加权:扩展])  #7 "系统评价(主题)"[不加权:扩展]  #8 "Meta分析"[不加权:扩展]  #9 "系统评价"[常用字段:智能] OR "meta分析"[常用字段:智能] OR "荟萃分析"[常用字段:智能] OR "元分析"[常用字段:智能]  #10 (#1) OR (#2)  #11 (#3) OR (#4)  #12 (#5) OR (#6)  #13 (#7) OR (#8) OR (#9)  #14 (#10) AND (#11) AND (#12) AND (#13) | April 1, 2024 |
| 维普 | 检索条件：((((((((题名或关键词=脑卒中 OR 题名或关键词=脑出血) OR 题名或关键词=脑血管意外) OR 题名或关键词=脑梗塞) OR 题名或关键词=卒中) OR 题名或关键词=中风) AND (((((((题名或关键词=经颅磁刺激 OR 题名或关键词=重复经颅磁刺激) OR 题名或关键词=磁刺激) OR 题名或关键词=rTMS) OR 题名或关键词=TMS) OR 题名或关键词=TBS) OR 题名或关键词=iTBS) OR 题名或关键词=cTBS) OR 题名或关键词=连续性θ节律性磁刺激) OR 题名或关键词=间歇性θ节律刺激) OR 题名或关键词=θ刺激) OR 题名或关键词=theta节律刺激) OR 题名或关键词=非入侵性脑刺激)) AND (((((题名或关键词=上肢 OR 题名或关键词=手) OR 题名或关键词=上肢运动功能) OR 题名或关键词=运动) OR 题名或关键词=手灵活性) OR 题名或关键词=痉挛)) AND (((题名或关键词=系统评价 OR 题名或关键词=meta分析) OR 题名或关键词=荟萃分析) OR 题名或关键词=元分析)) | April 1, 2024 |

**Supplementary Table S3.　List of included and excluded literature.**

| Serial number | Citation | included | excluded | reason |
| --- | --- | --- | --- | --- |
| 1 | Lerín‐Calvo A, Rodríguez‐Martínez D, Bernal‐Jiménez J J, et al. Combination of different noninvasive brain stimulation treatments for upper limb recovery in stroke patients: A systematic review[J]. Brain and Behavior, 2024, 14(1): e3370. |  | x | rTMS+tDCS |
| 2 | Abdullahi A, Wong T W L, Van Criekinge T, et al. Combination of noninvasive brain stimulation and constraint-induced movement therapy in patients with stroke: a systematic review and meta-analysis[J]. Expert Review of Neurotherapeutics, 2023, 23(2): 187-203. |  | x | protocol |
| 3 | Chen G, Lin T, Wu M, et al. Effects of repetitive transcranial magnetic stimulation on upper-limb and finger function in stroke patients: a systematic review and meta-analysis of randomized controlled trials[J]. Frontiers in neurology, 2022, 13: 940467. | x |  |  |
| 4 | Chen G, Wu M, Lin T, et al. Effects of repetitive transcranial magnetic stimulation on sequelae in patients with chronic stroke: A systematic review and meta-analysis of randomized controlled trials[J]. Frontiers in Neuroscience, 2022, 16: 998820. | x |  |  |
| 5 | Chen S, Zhang S, Yang W, et al. The effectiveness of intermittent theta burst stimulation for upper limb motor recovery after stroke: a systematic review and meta-analysis of randomized controlled trials[J]. Frontiers in Neuroscience, 2023, 17: 1272003. |  | x | includes other  interventions |
| 6 | Graef P, Dadalt M L R, da Silva Rodrigués D A M, et al. Transcranial magnetic stimulation combined with upper-limb training for improving function after stroke: a systematic review and meta-analysis[J]. Journal of the neurological sciences, 2016, 369: 149-158. |  | x | control group  containing rTMS |
| 7 | Duan X, Huang D, Zhong H, et al. Efficacy of rTMS in treating functional impairment in post-stroke patients: a systematic review and meta-analysis[J]. Neurological Sciences, 2024: 1-13. |  | x | rTMS+tDCS |
| 8 | Huang W, Chen J, Zheng Y, et al. The effectiveness of intermittent Theta burst stimulation for stroke patients with upper limb impairments: a systematic review and Meta-analysis[J]. Frontiers in Neurology, 2022, 13: 896651. |  | x | control group  containing rTMS |
| 9 | Narayan S K, Jayan J, Arumugam M. Short-term Effect of Noninvasive Brain Stimulation Techniques on Motor Impairment in Chronic Ischemic Stroke: A Systematic Review with Meta-Analysis[J]. Neurology India, 2022, 70(1): 37-49. |  | x | included study ＜5 |
| 10 | Naro A, Calabrò R S. Improving Upper Limb and Gait Rehabilitation Outcomes in Post-Stroke Patients: A Scoping Review on the Additional Effects of Non-Invasive Brain Stimulation When Combined with Robot-Aided Rehabilitation[J]. Brain Sciences, 2022, 12(11): 1511. |  | x | included study ＜5 |
| 11 | Tang I N. The effect of repetitive transcranial magnetic stimulation on upper extremity motor function in stroke patients: a meta-analytical review[J]. Journal of Food and Drug Analysis, 2012, 20(1): 4. |  | x | incomplete data |
| 12 | Tang Z, Han K, Wang R, et al. Excitatory repetitive transcranial magnetic stimulation over the ipsilesional hemisphere for upper limb motor function after stroke: a systematic review and meta-analysis[J]. Frontiers in Neurology, 2022, 13: 918597. | x |  |  |
| 13 | Zhao Q, Li H, Liu Y, et al. Non-invasive brain stimulation associated mirror therapy for upper-limb rehabilitation after stroke: Systematic review and meta-analysis of randomized clinical trials[J]. Frontiers in Neurology, 2022, 13: 918956. |  | x | includes other  interventions |
| 14 | Cha T H, Hwang H S. Rehabilitation interventions combined with noninvasive brain stimulation on upper limb motor function in stroke patients[J]. Brain Sciences, 2022, 12(8): 994. |  | x | incomplete data |
| 15 | Fan J, Fu H, Xie X, et al. The effectiveness and safety of repetitive transcranial magnetic stimulation on spasticity after upper motor neuron injury: A systematic review and meta-analysis[J]. Frontiers in Neural Circuits, 2022, 16: 973561. |  | x | other population |
| 16 | Hsu W Y, Cheng C H, Liao K K, et al. Effects of repetitive transcranial magnetic stimulation on motor functions in patients with stroke: a meta-analysis[J]. Stroke, 2012, 43(7): 1849-1857. | x |  |  |
| 17 | Le Q, Qu Y, Tao Y, et al. Effects of repetitive transcranial magnetic stimulation on hand function recovery and excitability of the motor cortex after stroke: a meta-analysis[J]. American journal of physical medicine & rehabilitation, 2014, 93(5): 422-430. | x |  |  |
| 18 | Le Q, Qu Y, Zhu S, et al. Meta-analysis of the effect of low-frequency repetitive transcranial magnetic stimulation on paretic hand recovery after stroke[J]. Sheng wu yi xue Gong Cheng xue za zhi= Journal of Biomedical Engineering= Shengwu Yixue Gongchengxue Zazhi, 2013, 30(6): 1229-1234. |  | x | full text not available |
| 19 | Lüdemann-Podubecká J, Bösl K, Nowak D A. Repetitive transcranial magnetic stimulation for motor recovery of the upper limb after stroke[J]. Progress in brain research, 2015, 218: 281-311. |  | x | rTMS+tDCS |
| 20 | McIntyre A, Mirkowski M, Thompson S, et al. A Systematic Review and Meta‐Analysis on the Use of Repetitive Transcranial Magnetic Stimulation for Spasticity Poststroke[J]. PM&R, 2018, 10(3): 293-302. |  | x | incomplete data |
| 21 | O'Brien A T, Bertolucci F, Torrealba‐Acosta G, et al. Non‐invasive brain stimulation for fine motor improvement after stroke: a meta‐analysis[J]. European journal of neurology, 2018, 25(8): 1017-1026. |  | x | other population |
| 22 | O'Brien A T, Acosta G T, Huerta R, et al. Does non-invasive brain stimulation modify hand dexterity? Protocol for a systematic review and meta-analysis[J]. BMJ open, 2017, 7(6): e015669. |  | x | Protocol |
| 23 | Reis S B, Bernardo W M, Oshiro C A, et al. Effects of robotic therapy associated with noninvasive brain stimulation on upper-limb rehabilitation after stroke: systematic review and meta-analysis of randomized clinical trials[J]. Neurorehabilitation and Neural Repair, 2021, 35(3): 256-266. |  | x | non-rTMS |
| 24 | Safdar A, Smith M C, Byblow W D, et al. Applications of repetitive transcranial magnetic stimulation to improve upper limb motor performance after stroke: a systematic review[J]. Neurorehabilitation and Neural Repair, 2023, 37(11-12): 837-849. |  | x | rTMS+tDCS |
| 25 | Sanchez-Cuesta F J, Gonzalez-Zamorano Y, Arroyo-Ferrer A, et al. Repetitive transcranial magnetic stimulation of primary motor cortex for stroke upper limb motor sequelae rehabilitation: A systematic review[J]. NeuroRehabilitation, 2023 (Preprint): 1-20. |  | x | includes other  interventions |
| 26 | Vabalaite B, Petruseviciene L, Savickas R, et al. Effects of high-frequency (HF) repetitive transcranial magnetic stimulation (rTMS) on upper extremity motor function in stroke patients: A systematic review[J]. Medicina, 2021, 57(11): 1215. |  | x | included study ＜5 |
| 27 | Xi X, Wang H, Han L, et al. Meta-analysis of repetitive transcranial magnetic stimulation combined with task-oriented training on upper limb function in stroke patients with hemiplegia[J]. Medicine, 2023, 102(22): e33771. |  | x | control group  containing rTMS |
| 28 | Zhang L, Xing G, Fan Y, et al. Short-and long-term effects of repetitive transcranial magnetic stimulation on upper limb motor function after stroke: a systematic review and meta-analysis[J]. Clinical rehabilitation, 2017, 31(9): 1137-1153. |  | x | relevant indicators  not included |
| 29 | Zhang, Lan et al. “Low-Frequency Repetitive Transcranial Magnetic Stimulation for Stroke-Induced Upper Limb Motor Deficit: A Meta-Analysis.” Neural plasticity vol. 2017 (2017): 2758097. doi:10.1155/2017/2758097 | x |  |  |
| 30 | Alashram A R, Padua E, Romagnoli C, et al. Effects of Repetitive transcranial magnetic stimulation on Upper extremity spasticity Post-Stroke: A Systematic review[J]. Physikalische Medizin, Rehabilitationsmedizin, Kurortmedizin, 2022, 32(03): 136-145. | x |  |  |
| 31 | Fisicaro F, Lanza G, Grasso A A, et al. Repetitive transcranial magnetic stimulation in stroke rehabilitation: review of the current evidence and pitfalls[J]. Therapeutic advances in neurological disorders, 2019, 12: 1756286419878317. |  | x | relevant indicators  not included |
| 32 | He Y, Li K, Chen Q, et al. Repetitive transcranial magnetic stimulation on motor recovery for patients with stroke: a PRISMA compliant systematic review and meta-analysis[J]. American Journal of Physical Medicine & Rehabilitation, 2020, 99(2): 99-108. | x |  |  |
| 33 | Jiang T, Wei X, Wang M, et al. Theta burst stimulation: what role does it play in stroke rehabilitation? A systematic review of the existing evidence[J]. BMC neurology, 2024, 24(1): 52. | x |  |  |
| 34 | Jin Y, Pu T, Guo Z, et al. Placebo effect of rTMS on post-stroke motor rehabilitation: a meta-analysis[J]. Acta Neurologica Belgica, 2021, 121(4): 993-999. |  | x | relevant indicators  not included |
| 35 | Liu X, Zhong J, Xiao X, et al. Theta burst stimulation for upper limb motor dysfunction in patients with stroke: A protocol of systematic review and meta-analysis[J]. Medicine, 2019, 98(46): e17929. |  | x | protocol |
| 36 | Nanji L S, Cardoso A T, Costa J, et al. Analysis of the Cochrane Review: Interventions for Improving Upper Limb Function after Stroke. Cochrane Database Syst Rev. 2014, 11: CD010820[J]. Acta medica portuguesa, 2015, 28(5): 551-553. |  | x | language |
| 37 | Sebastianelli L, Versace V, Martignago S, et al. Low‐frequency rTMS of the unaffected hemisphere in stroke patients: A systematic review[J]. Acta Neurologica Scandinavica, 2017, 136(6): 585-605. |  | x | relevant indicators  not included |
| 38 | Subramanian S K, Prasanna S S. Virtual Reality and noninvasive brain stimulation in stroke: how effective is their combination for upper limb motor improvement?—a meta-analysis[J]. PM&R, 2018, 10(11): 1261-1270. |  | x | non-rTMS |
| 39 | van Lieshout E C C, van der Worp H B, Visser-Meily J M A, et al. Timing of repetitive transcranial magnetic stimulation onset for upper limb function after stroke: a systematic review and meta-analysis[J]. Frontiers in neurology, 2019, 10: 1269. | x |  |  |
| 40 | Wang X, Ge L, Hu H, et al. Effects of non-invasive brain stimulation on post-stroke spasticity: a systematic review and meta-analysis of randomized controlled trials[J]. Brain Sciences, 2022, 12(7): 836. | x |  |  |
| 41 | Xiang H, Sun J, Tang X, et al. The effect and optimal parameters of repetitive transcranial magnetic stimulation on motor recovery in stroke patients: a systematic review and meta-analysis of randomized controlled trials[J]. Clinical rehabilitation, 2019, 33(5): 847-864. |  | x | other population |
| 42 | Xu P, Huang Y, Wang J, et al. Repetitive transcranial magnetic stimulation as an alternative therapy for stroke with spasticity: a systematic review and meta-analysis[J]. Journal of Neurology, 2021, 268: 4013-4022. |  | x | incomplete data |
| 43 | Gao YL. Effect of repetitive transcranial magnetic stimulation for upper  extremity dysfunction after stroke[D]. Dalian Medical University, 2021. | x |  |  |
| 44 | Huang GL, Tang XL, Huang Y. Meta-analysis of the effects of 1Hz low-frequency repetitive transcranial magnetic stimulation on upper limb spasticity and motor function in hemiplegia after strok[J].China journal rehabilitation medicine, 2018, 33(6): 701-705. |  | x | control group  containing rTMS |
| 45 | Le Q, Qu Y, Zhhu SJ, et al. Meta-analysis of the Effect of Low-frequency Repetitive TranscranialMagnetic Stimulation on Paretic Hand Recovery after Stroke[J]. Journal of Biomedical Engineering, 2013, 30(6): 1229-1234. | x |  |  |
| 46 | Li JX, Zhang L, Guo YL, et al. A Meta-Analysis of the Effect of 10Hz-rTMS on Upper Limb Motor Function Ｒehabilitation in Stroke Patients[J]. Hebei Medicine, 2022, 28(10): 1712-1719. |  | x | incomplete data |
| 47 | Ling HM, Tao T, XU J, et al. Effects of repetitive transcranial magnetic stimulation on upper limb motor function in patients with stroke:a meta analysis[J]. National Medical Journal of China, 2017, 97(47): 3739-3745. | x |  |  |
| 48 | Wang J, Zhu F, Wang P, et al. Eff ectiveness of Repetitive Transcranial Magnetic Stimulation in Stroke Patients with Motor Dysfunction: A Systematic Review[J].中Chinese Journal of Evidence-Based Medicine, 2012, 12(12): 1478-1488. |  | x | relevant indicators  not included |
| 49 | Wu M, Li ZN, Liu SY, et al. Meta-analysis of the efficacy of repetitive transcranial magnetic stimulation on the rehabilitation of hand function in stroke patients[J]. Chinese Journal of Rehabilitation Medicine, 2022, 37(03): 372-376. | x |  |  |
| 50 | Xia Y, Lu Y, Li AL, et al. Effects of theta burst stimulation on upper limb motor function and activities of daily living in patients after stroke: a Meta analysis[J].Chinese Journal of Medical Physics, 2022, 39(03): 341-348. | x |  |  |
| 51 | Yang F, Liu L, Guo BB, et al. Effectiveness and safety of repetitive transcranial magnetic stimulation in patients with post-stroke motor dysfunction: a meta-analysis[J]. Chinese Journal of Cerebrovascular Diseases, 2012, 9(06): 284-290. |  | x | relevant indicators  not included |
| 52 | Zhu Y, Yang YJ, Gu YH, et al. Efficiency of repetitive transcranial magnetic stimulation on rehabilitation of motor function in patients with stroke: A systematic review[J]. Chinese Journal of Tissue Engineering Research, 2013, 17(50): 8758-8768. |  | x | relevant indicators  not included |
| 53 | He YJ. REPETITIVE TRANSCRANIAL MAGNETIC STIMULATION ON MOTOR RECOVERY FOR PATIENTS WITH STROKE: A PRISMA COMPLIANT SYSTEMATIC REVIEW AND META-ANALYSIS[D]. Chongqing Medical University, 2019. |  | x | incomplete data |
| 54 | Liu A. The Effects of Transcranial Magnetic Stimulation iSpasticity following upper motor neuroneimpairment:A Systematic Review[D]. Fujian University of Chinese Medicine, 2015. |  | x | other population |
| 55 | Alashram A R. Non-Invasive Brain Stimulation Combined with Neuromuscular Electrical Stimulation for Upper Limb Rehabilitation in Stroke Survivors: A Systematic Review[J]. Current Physical Medicine and Rehabilitation Reports, 2024: 1-19. |  | x | included study ＜5 |
| 56 | Ahmed I, Mustafaoglu R, Benkhalifa N, et al. Does noninvasive brain stimulation combined with other therapies improve upper extremity motor impairment, functional performance, and participation in activities of daily living after stroke? A systematic review and meta-analysis of randomized controlled trial[J]. Topics in Stroke Rehabilitation, 2023, 30(3): 213-234. |  | x | includes other  interventions |
| 57 | Xie Y, Pan J H, Chen J, et al. Acupuncture combined with repeated transcranial magnetic stimulation for upper limb motor function after stroke: a systematic review and meta-analysis[J]. NeuroRehabilitation, 2023, 53(4): 423-438. |  | x | control group  containing rTMS |
| 58 | Zhi J F, Liao Q H, He Y B, et al. Superior treatment efficacy of neuromodulation rehabilitation for upper limb recovery after stroke: a meta-analysis[J]. Expert Review of Neurotherapeutics, 2022, 22(10): 875-888. |  | x | includes other  interventions |
| 59 | Alashram A R. Combined noninvasive brain stimulation virtual reality for upper limb rehabilitation poststroke: A systematic review of randomized controlled trials[J]. Neurological Sciences, 2024: 1-15. |  | x | included study ＜5 |
| 60 | Gao B, Wang Y, Zhang D, et al. Intermittent theta-burst stimulation with physical exercise improves poststroke motor function: A systemic review and meta-analysis[J]. Frontiers in neurology, 2022, 13: 964627. | x |  |  |
| 61 | Ling HM. Eeffects of Repetitive Transcranial Magnetic Stimulation on Upper Limb Motor Function in Patients With Stroke: A Meta Analysis[D]. Guizhou Medical University, 2016. |  | x | duplicate  publications |

**Supplementary Table S4.　Characteristics of the included MAs/SRs.**

| Study ID | Country | Stroke type | Search time | Age (years) | Research type | Trials/n | Experimental group | Control group | Basic features of rTMS | Methodology evaluation tools | Outcomes | Adverse events | Conflict of interest | Sources of funding | Meta-analysis | Main conclusion |
| --- | --- | --- | --- | --- | --- | --- | --- | --- | --- | --- | --- | --- | --- | --- | --- | --- |
| Le et al. (2013) | China | Mixed | Feb 1990 to Apr 2012 | ≥18 | RCTs | 7/216 | LF-rTMS+m edication+rehabilitation | Medication +rehabilitation | M1; 1/3/5 Hz;10%-130% rMT; 2.5-20 min/session; 1-10 days | PEDro scale | PPT, FT, JTT, mRS | Mild headaches, anxiety | Not reported | Not reported | Yes | The evidence showed that low-frequency rTMS could improve the paretic hand recovery after stroke |
| Ling et al. (2017) | China | Mixed | To Oct 2015 | ＞18 | RCTs | 9/289 | rTMS/iTBS+medication+rehabilitation | Sham; sham+medication+rehabilitation | 1/3/5/10/20/50 Hz; 5 days-4 weeks | Cochrane risk of bias tool | WMFT, FT, KT | Mild headaches, anxiety | Not reported | Not reported | Yes | The meta analysis suggests that rTMS has a positive effect on motor recovery in patients with stroke, especially the low frequency rTMS may be more beneficial. The clinical application of rTMS in patients with stroke is relatively safe.Intermittent theta-burst stimulation might be a useful intervention |
| Wu et al. (2022) | China | Mixed | To May 2020 | ＞18 | RCTs | 8/290 | rTMS; rTMS+PT/medication/routine treatment/group model training | Sham; task-oriented training/PT/medication/upper limb motor training | 1/3Hz; 50%-100% MT; 420-1200 pulses; 20-30 min/session, 3-5 sesstions/weeks, 2-8 weeks | Cochrane risk of bias tool, new Castle Ottawa scale | FMA-UE, MBI, MAS, Brunnstrom assessment of hand motor function | Not reported | Not reported | Not industrial | Yes | rTMS to improve hand motor function cannot yet be confirmed. Given the average quality of the included literature, further high quality clinical double-blind randomized controlled trials with multicenter, large samples and long-term observations are needed |
| Xia et al. (2022) | China | Mixed | To Apr 2021 | ＞18 | RCTs | 14/329 | TBS+routine treatment | Sham+routine treatment | 30/50 Hz; 600/1200/2000 pulses; 10-15 sessions | Cochrane risk of bias tool | ARAT, FMA-UE, NIHSS, MBI | Not reported | Not reported | Not industrial | Yes | TBS can effectively improve upper limb motor function and activities of daily living in stroke patients, but the above conclusion still needs to be further confirmed by more high-quality researches |

**Supplementary Table S4.　(continued).**

| Study ID | Country | Stroke type | Search time | Age (years) | Research type | Trials/n | Experimental group | Control group | Basic features of rTMS | Methodology evaluation tools | Outcomes | Adverse events | Conflict of interest | Sources of funding | Meta-analysis | Main conclusion |
| --- | --- | --- | --- | --- | --- | --- | --- | --- | --- | --- | --- | --- | --- | --- | --- | --- |
| Gao (2021) | China | Mixed | To Feb 2021 | ≥18 | RCTs, crossover RCTs | 11/444 | rTMS+medication/routine treatment | Sham/medication/routine treatment | Not reported | Cochrane risk of bias tool | FMA-UE, MAS, MBI, BI, MEP latency, adverse events rate | No serious adverse events | Not reported | Not reported | Yes | rTMS and TBS improved upper extremity function after stroke, but efficacy in upper extremity spasticity was not determined. rTMS combined regimen was applied with a relatively good clinical safety profile |
| Hsu et al. (2012) | China, Taiwan | Mixed | Jan 1990 to Oct 2011 | ＞18 | RCTs | 18/392 | rTMS; rTMS+exercise therapy/CIMT/VMC/EMG-FNMS | Sham; sham+exercise therapy/CIMT/VMC/EMG-FNMS | UH/AH M1; 1/3/10/30/50 Hz; M1; 80-130% MT; 600-1200 pulses | Modified checklist derived from a quality screening form | MT | Headahes, anxiety, fatigue | Not reported | Not industrial | Yes | rTMS has a positive effect on motor recovery in patients with stroke, especially for those with subcortical stroke. Low-frequency rTMS over the unaffected hemisphere may be more beneficial than high-frequency rTMS over the affected hemisphere |
| Chen et al. (2022a) | China | Mixed | To Feb 12, 2022 | ≥18 | RCTs | 45/2, 064 | rTMS; rTMS+other therapies | Sham/other therapies | 1/3/5/10/20/30/50 Hz; 80-120% MT; 500-1800 pulses; 5-20 sessions | PEDro scale | FMA-UE, BBT, PPT, NHPT | Not reported | No | Not industrial | Yes | This updated meta-analysis provides robust evidence of the efficacy of rTMS treatment in improving upper extremity and fine function during various phases of stroke |
| Tang et al. (2022) | China | Mixed | To Dec 31, 2021 | ≥18 | RCTs | 15/449 | rTMS/iTBS | Sham/routine treatment | M1; 3/5/10/30 Hz; 80-120% aMT/80% MT; 600-2000 pulses; 5-20 sessions | PEDro scale | FMA-UE, PF, GS, ARAT, BBT, JTT, WMFT, NHPT, MEP amplitude | Transient headaches, tingling sensations on the head, numbness in the scalp and facial muscles, seizures, increased paroxysmal or newly emerged epileptiform EEG activity, lower extremity deep vein thrombosis and thrombus flotation | No | Not industrial | Yes | The study demonstrated that excitatory rTMS over the ipsilesional hemisphere could significantly improve upper limb motor function, hand strength, and hand dexterity in patients diagnosed with stroke. Both iTBS and HF-rTMS which could significantly promote upper limb motor function and hand dexterity, and excitatory rTMS were beneficial to upper limb motor function recovery only when applied in the first 3 months after stroke. HF-rTMS could significantly enhance the MEP amplitude of the affected hemisphere. High-quality and large-scale randomized controlled trials in the future are required to confirm our conclusions |

**Supplementary Table S4.　(continued).**

| Study ID | Country | Stroke type | Search time | Age (years) | Research type | Trials/n | Experimental group | Control group | Basic features of rTMS | Methodology evaluation tools | Outcomes | Adverse events | Conflict of interest | Sources of funding | Meta-analysis | Main conclusion |
| --- | --- | --- | --- | --- | --- | --- | --- | --- | --- | --- | --- | --- | --- | --- | --- | --- |
| Zhang et al. (2017) | China | Mixed | To Jun 31, 2017 | ≥18 | RCTs | 22/619 | LF-rTMS+exercise therapy/passive movement/PT/extensor activity/rehabilitation/Task-oriented training/OT/functional task practice | Sham; sham+exercise therapy/passive movement/PT/extensor activity/rehabilitation/Task-oriented training/OT/functional task practice | UH M1; 1 Hz; 90-120% rMT; 600-1800 pulses; 1-20 days | Modified checklist derived from a quality screening form | rMT, MEP, PF, GS, NHPT, FT, WMFT, FMA-UE, ARAT | Not reported | No | Not industrial | Yes | LF-rTMS as an add-on therapy significantly improved upper limb functional recovery especially the hand after stroke, probably through rebalanced cortical excitability of both hemispheres. Future studies should determine if LF-rTMS alone or in conjunction with practice/training would be more effective |
| Le et al. (2014) | China | Mixed | To Jan 30, 2012 | ≥18 | RCTs | 8/273 | rTMS | placebo/other therapies | M1; 1/3/5/10/20/25 Hz; 80-130% rMT; 1-10 days | PEDro scale | PPT, PF, FT, mRS, JTT, WMFT, MEP amplitude, aMT | Headahe, anxiety, tingling sensation | Not reported | Not reported | Yes | rTMS can improve patients’ recovery after stroke. Future trials can concentrate on the effects of rTMS for different types of stroke patients in response to stimulation at different sites and explore optimal rTMS parameters for individual treatment |
| Alashram et al. (2022) | Jordan | Mixed | To Jun 2021 | No limit | RCTs | 10/225 | rTMS/iTBS; iTBS+PT; rTMS+PT | Sham; Sham+PT | M1/UH/AH; 1/10 Hz; 80-100% rMT; 240-1500 pulses; 10-20 sessions | Cochrane risk of bias tool | MAS | Seizures and increased epileptiform activity, EEG revealed an increase in epilep f tiform activity | No | Not reported | No | Combining rTMS with other rehabilitation interventions may show a superior effect in reducing the upper extremity spasticity compared with rTMS intervention alone. Further randomized controlled trials with long-term follow-ups are warranted |

**Supplementary Table S4.　(continued).**

| Study ID | Country | Stroke type | Search time | Age (years) | Research type | Trials/n | Experimental group | Control group | Basic features of rTMS | Methodology evaluation tools | Outcomes | Adverse events | Conflict of interest | Sources of funding | Meta-analysis | Main conclusion |
| --- | --- | --- | --- | --- | --- | --- | --- | --- | --- | --- | --- | --- | --- | --- | --- | --- |
| He et al. (2020) | China | Mixed | To Sep 20, 2018 | ≥18 | RCTs | 20/598 | rTMS; rTMS+PT+OT/PT/BHM/comprehensive rehabilitation therapy/VR training+comprehensive rehabilitation therapy/routine treatment+medication/routine treatment | PT+OT/PT/sham /routine treatment; sham+BHM/routine treatment/routine treatment+medication | 1/3/10 Hz; 70-130% MT; 600-1800 pulses; 1.25-25 min/sesstion; 1 week-12 months | Cochrane risk of bias tool | FMA-UE, MBI, BI, JTT, GS, NIHSS | Dizziness, abnormal sleep, nausea, nonspecific neck pain | No | Not reported | Yes | The analysis showed that low-frequency repetitive transcranial magnetic stimulation has a positive effect on grip strength and lower limb function as assessed by FMA |
| van Lieshout et al. (2019) | Netherlands | Mixed | To Feb 2018 | ≥18 | RCTs | 38/1, 074 | rTMS/iTBS; rTMS+PT/OT/upper limb motor training/routine treatment/motor learning task/functional task practice/medication/BHM+upper limb motor training/voluntary muscle contraction/exerciae therapy/EMG-FNMS/reach-to-grasp training/VR training | Sham; sham+PT/OT/upper limb motor training/routine treatment/motor learning task/functional task practice/medication/hand manipulation+upper limb motor training/voluntary muscle contraction/exerciae therapy/EMG-FNMS/reach-to-grasp training/VR training | AH M1/UH M1/right hemisphere, P3 10/20 EEG system/; 1/5/20 Hz; 80-130% rMT; 600-1800 pulses | Cochrane risk of bias tool | RT, FMA-UE, FT, WMFT, ARAT, JTT, GS, PF, BBT | Not reported | No | Not industrial | Yes | Based on the FMA, rTMS seems more beneficial only when started in the first month post-stroke. Tests at the level of function are likely more sensitive to detect beneficial rTMS effects on upper limb function than tests at the level of activity. However, heterogeneities in treatment designs and outcomes are high. Future rTMS trials should include the FMA and work toward a core set of outcome measures |
| Chen et al. (2022b) | China | chronic | To Sep 11, 2022 | No limit | RCTs | 15/227 | rTMS; rTMS+other therapies | Sham/other therapies | AH M1/UH M1/Bilateral M1/Left DLPFC; 1/5/10/20/30/50 Hz; 600-2000 pulses; 80-90% aMT/70-100% rMT/90-110% MT; 1-20 sessions | PEDro scale | BBT, PPT, NHPT | Mild headaches, anxiety, mild neck pain, mild sleep disturbance, scalp discomfort | No | Not industrial | Yes | This is the first meta-analysis of rTMS treatment in patients with chronic stroke to inform the selection of the optimal treatment strategy for patients with chronic stroke, which demonstrated that rTMS treatment has the potential to improve the effects of sequelae by improving upper limb function,hand function,and muscle tone |

**Supplementary Table S4.　(continued).**

| Study ID | Country | Stroke type | Search time | Age (years) | Research type | Trials/n | Experimental group | Control group | Basic features of rTMS | Methodology evaluation tools | Outcomes | Adverse events | Conflict of interest | Sources of funding | Meta-analysis | Main conclusion |
| --- | --- | --- | --- | --- | --- | --- | --- | --- | --- | --- | --- | --- | --- | --- | --- | --- |
| Jiang et al. (2024) | China | Mixed | To Aug 2023 | No limit | RCTs | 12/318 | TBS+PT+OT/routine treatment/VCT/robot-assisted training | sham; PT+OT/routine treatment/VCT/robot-assisted training | 600 pulses; 70-80% rMT; 1-2 sessions/day;10 days-4 weeks | Cochrane risk of bias tool | MAS, NHPT, ARAT, FMA-UE | Not reported | No | No | Yes | Over all, TBS promotes the progress of stroke rehabilitation and may serve as a preferable alternative to traditional rTMS. However, it’s hard to recommend a specifc paradigm of TBS due to the limited number of current studies and their heterogeneity. Further high-quality clinical RCTs are needed to determine the optimal technical settings and interven tion time in stroke survivors |
| Wang et al. (2022) | China | Mixed | To Dec 2021 | No limit | RCTs | 14/232 | rTMS/TBS; iTBS+VCT/rTMS+PT/rTMS+rehabilitation | Sham; sham+VCT/rehabilitation | 1/50 Hz; 200-1500 pulses; 80-100% rMT | Cochrane risk of bias tool | MAS | Not reported | No | Not industrial | Yes | This meta-analysis revealed moderate evidence that NIBS reduces spasticity after stroke and may promote recovery in stroke survivors. Future studies investigating the mechanisms of NIBS in addressing spasticity are warranted to further support the clinical application of NIBS in post-stroke spasticity |
| Gao et al. (2022) | China | Mixed | To May 2022 | No limit | RCTs | 9/397 | TBS+robot-assisted training/PT/PT+OT/medication+rehabilitation/routinr treatment/VCT | Sham; sham+robot-assisted training/PT/PT+OT/medication+rehabilitation/routinr treatment/VCT | Ipsilesional M1/FDI hot spot/ ipsilesional lateral cerebellum/the hand motor area of the AH; 600/1200 pulses; 10 days-4 weeks | Cochrane risk of bias tool | ARAT, MAS, WMFT | Not reported | No | Not industrial | Yes | This study supports that iTBS has good efficacy for improving motor function in stroke patients. Therefore, standard 600-pulse stimulation iTBS therapy is proper management and treatment for chronic stroke |

LF-rTMS, Low frequency rTMS; HF-rTMS, High frequency rTMS; UH, Unaffected hemisphere; AH, affected hemisphere; M1, primary motor cortex M1 area; DLPFC, dorsolateral prefrontal cortex; FDI, first dorsal interosseous; FMA, Fugl-Meyer Assessment.

**Supplementary Table S5.　AMSTAR-2 scale included MAs/SRs.**

| Study ID | 1 | 2* | 3 | 4* | 5 | 6 | 7* | 8 | 9* | 10 | 11* | 12 | 13* | 14 | 15* | 16 | Quality grade |
| --- | --- | --- | --- | --- | --- | --- | --- | --- | --- | --- | --- | --- | --- | --- | --- | --- | --- |
| Le et al. (2013) | Y | N | N | PY | N | N | N | Y | Y | N | N | N | N | N | N | N | Very low |
| Ling et al. (2017) | Y | N | N | PY | Y | Y | N | Y | Y | N | Y | Y | Y | Y | N | N | Very low |
| Wu et al. (2022) | Y | N | N | PY | Y | N | N | Y | Y | N | N | N | N | N | N | N | Very low |
| Xia et al. (2022) | Y | N | N | PY | Y | Y | N | Y | Y | N | Y | Y | Y | Y | Y | N | Very low |
| Gao (2021) | Y | N | N | PY | Y | Y | N | PY | Y | N | Y | Y | Y | Y | Y | N | Very low |
| Hsu et al. (2012) | Y | N | N | PY | Y | Y | N | Y | Y | N | Y | N | N | Y | Y | N | Very low |
| Chen et al. (2022a) | Y | PY | N | PY | N | Y | N | Y | Y | N | Y | N | Y | Y | N | Y | Very low |
| Tang et al. (2022) | Y | PY | N | PY | Y | Y | N | Y | Y | N | Y | N | N | N | N | N | Very low |
| Zhang et al. (2017) | Y | PY | N | PY | Y | Y | N | Y | PY | N | Y | N | Y | N | Y | Y | Very low |
| Le et al. (2014) | N | N | N | PY | N | Y | N | PY | Y | N | Y | N | N | N | N | N | Very low |
| Alashram et al. (2022) | Y | N | N | PY | Y | Y | N | Y | Y | N | NM | NM | N | N | N | N | Very low |
| He et al. (2020) | Y | PY | N | PY | N | Y | N | Y | Y | N | N | Y | Y | Y | Y | N | Very low |
| van Lieshout et al. (2019) | Y | N | N | PY | Y | Y | N | Y | Y | Y | N | N | N | N | Y | N | Very low |
| Chen et al. (2022b) | Y | PY | N | PY | N | Y | N | PY | Y | N | N | N | N | Y | N | Y | Very low |
| Jiang et al. (2024) | Y | PY | N | PY | Y | Y | N | Y | Y | N | Y | N | N | Y | N | Y | Very low |
| Wang et al. (2022) | Y | N | N | PY | Y | N | N | PY | Y | N | N | N | N | Y | N | Y | Very low |
| Gao et al. (2022) | Y | N | N | PY | Y | Y | N | PY | Y | N | N | Y | Y | Y | N | Y | Very low |
| Reporting rate (%) | 94.1 | 35.3 | 0 | 100 | 70.6 | 82.4 | 0 | 100 | 100 | 5.9 | 52.9 | 29.4 | 41.2 | 58.8 | 35.3 | 35.3 | — |

Entry1 Do the study questions and inclusion criteria include PICO? entry 2: Is there a pre-published protocol? Is there significant bias between the study and the protocol? entry 3: Did the authors explain the type of study design included? entry 4: Was a comprehensive literature search strategy used? entry 5: Was duplicate study screening performed? entry 6: Were duplicate data extractions performed? entry 7: Was a list of excluded literature provided, with reasons for the exclusion? entry 8: Was a detailed description of the included studies provided? entry 9: Was the risk of bias for each included study assessed using a reasonable tool? entry 10: Is the source of funds for the included studies reported? entry 11: If Meta-analyses were performed, were the results statistically combined using appropriate methods? entry 12: If Meta-analyses were performed, is the effect of risk of bias described in the results? entry 13: If a Meta-analysis was performed, is the effect of risk of bias described in the discussion? entry 14: Is heterogeneity justified in the discussion? entry15: If a quantitative analysis was performed, was publication bias adequately investigated and its possible impact discussed? entry 16: Are any potential sources of conflict of interest reported?

* represents the critical items in the AMSTAR 2 scale;Y = Yes; PY = Partial Yes; N = No; NM = No meta- analysis

Entry reporting rate = [(number of studies with full met of entries + number of studies with partial met)/total number of included studies] × 100%

**Supplementary Table S6.　PRISMA declaration entries report conditions.**

| Stud ID | 1 | 2 | 3 | 4 | 5 | 6 | 7 | 8 | 9 | 10 | 11 | 12 | 13 | 14 | 15 |
| --- | --- | --- | --- | --- | --- | --- | --- | --- | --- | --- | --- | --- | --- | --- | --- |
| Le et al. (2013) | 1 | 0.5 | 1 | 1 | 0.5 | 0.5 | 0 | 0 | 0 | 0.5 | 1 | 1 | 0.5 | 1 | 0 |
| Ling et al. (2017) | 1 | 0.5 | 1 | 1 | 0.5 | 0.5 | 0 | 1 | 1 | 0.5 | 1 | 1 | 1 | 1 | 0 |
| Wu et al. (2022) | 1 | 0 | 1 | 1 | 1 | 0.5 | 0.5 | 1 | 0.5 | 0.5 | 0.5 | 0.5 | 0.5 | 0 | 0 |
| Xia et al. (2022) | 1 | 0.5 | 1 | 1 | 1 | 0.5 | 0.5 | 1 | 1 | 0.5 | 1 | 1 | 0.5 | 0 | 0 |
| Gao (2021) | 1 | 0.5 | 1 | 1 | 1 | 0.5 | 0.5 | 1 | 1 | 0.5 | 0.5 | 1 | 0.5 | 1 | 0 |
| Hsu et al. (2012) | 1 | 0.5 | 1 | 1 | 0.5 | 0.5 | 0 | 0 | 0.5 | 0.5 | 0.5 | 1 | 0.5 | 1 | 0 |
| Chen et al. (2022a) | 1 | 1 | 1 | 1 | 0.5 | 0.5 | 1 | 0 | 1 | 0.5 | 1 | 0.5 | 0.5 | 0 | 0 |
| Tang et al. (2022) | 1 | 1 | 1 | 1 | 1 | 0.5 | 1 | 1 | 1 | 0.5 | 1 | 1 | 0.5 | 0 | 0 |
| Zhang et al. (2017) | 1 | 1 | 1 | 1 | 0.5 | 0.5 | 0 | 1 | 0.5 | 0.5 | 0.5 | 1 | 0.5 | 1 | 0 |
| Le et al. (2014) | 1 | 0.5 | 1 | 1 | 0.5 | 0.5 | 0 | 0.5 | 0.5 | 0.5 | 1 | 1 | 0.5 | 1 | 0 |
| Alashram et al. (2022) | 1 | 0.5 | 1 | 1 | 0.5 | 0.5 | 0 | 1 | 0.5 | 0.5 | 1 | 0 | 0 | 0 | 0 |
| He et al. (2020) | 1 | 0.5 | 1 | 1 | 0.5 | 0.5 | 1 | 0 | 0.5 | 0.5 | 1 | 1 | 0.5 | 1 | 1 |
| van Lieshout et al. (2019) | 1 | 0.5 | 1 | 1 | 0.5 | 0.5 | 1 | 1 | 0.5 | 1 | 1 | 1 | 1 | 1 | 0 |
| Chen et al. (2022b) | 1 | 1 | 1 | 1 | 0.5 | 0.5 | 1 | 0 | 1 | 0.5 | 1 | 1 | 1 | 0 | 1 |
| Jiang et al. (2024) | 1 | 0.5 | 1 | 1 | 0.5 | 0.5 | 1 | 1 | 0.5 | 0.5 | 1 | 1 | 1 | 1 | 1 |
| Wang et al. (2022) | 1 | 0.5 | 1 | 1 | 0.5 | 0.5 | 0 | 1 | 0.5 | 0.5 | 1 | 1 | 0.5 | 1 | 0 |
| Gao et al. (2022) | 1 | 0.5 | 1 | 1 | 0.5 | 0.5 | 0 | 1 | 1 | 0.5 | 0.5 | 1 | 0.5 | 0 | 0 |
| Reporting rate (%) | 100 | 58.8 | 100 | 100 | 61.8 | 50 | 41.2 | 67.6 | 67.6 | 52.9 | 85.3 | 88.2 | 58.8 | 58.8 | 17.6 |

**Supplementary Table S6.　(continued).**

| Stud ID | 16 | 17 | 18 | 19 | 20 | 21 | 22 | 23 | 24 | 25 | 26 | 27 | Total Score |
| --- | --- | --- | --- | --- | --- | --- | --- | --- | --- | --- | --- | --- | --- |
| Le et al. (2013) | 0.5 | 0.5 | 1 | 1 | 0.5 | 0 | 0 | 1 | 0 | 0 | 0 | 0 | 13 |
| Ling et al. (2017) | 0.5 | 0.5 | 0.5 | 1 | 0.5 | 0 | 0 | 0.5 | 0 | 0 | 0 | 0 | 14.5 |
| Wu et al. (2022) | 0.5 | 1 | 1 | 1 | 0.5 | 0 | 0 | 0.5 | 0 | 0.5 | 0 | 0 | 13.5 |
| Xia et al. (2022) | 1 | 1 | 1 | 1 | 1 | 1 | 0 | 1 | 0 | 0.5 | 0 | 0 | 18 |
| Gao (2021) | 0.5 | 0.5 | 1 | 1 | 1 | 1 | 0 | 0.5 | 0 | 0 | 0 | 0 | 16.5 |
| Hsu et al. (2012) | 0.5 | 0.5 | 1 | 1 | 0.5 | 1 | 0 | 1 | 0 | 0.5 | 0 | 0 | 14.5 |
| Chen et al. (2022a) | 1 | 1 | 1 | 1 | 1 | 0 | 0 | 1 | 0.5 | 0.5 | 1 | 1 | 18.5 |
| Tang et al. (2022) | 1 | 0.5 | 1 | 1 | 0.5 | 0 | 0 | 1 | 0.5 | 0.5 | 1 | 1 | 19.5 |
| Zhang et al. (2017) | 0.5 | 1 | 1 | 1 | 0.5 | 1 | 0 | 1 | 0.5 | 0.5 | 1 | 1 | 19 |
| Le et al. (2014) | 0.5 | 1 | 1 | 1 | 0.5 | 0 | 0 | 1 | 0 | 0 | 1 | 0 | 15.5 |
| Alashram et al. (2022) | 0.5 | 1 | 1 | 0 | 0 | 0 | 0 | 1 | 0 | 0 | 1 | 0 | 12 |
| He et al. (2020) | 1 | 1 | 0 | 1 | 1 | 1 | 1 | 1 | 0.5 | 0 | 1 | 1 | 20.5 |
| van Lieshout et al. (2019) | 1 | 1 | 1 | 1 | 0.5 | 1 | 0 | 1 | 0 | 0.5 | 0 | 1 | 20 |
| Chen et al. (2022b) | 1 | 0.5 | 1 | 1 | 1 | 0 | 1 | 1 | 0.5 | 0.5 | 1 | 1 | 21 |
| Jiang et al. (2024) | 1 | 0.5 | 1 | 1 | 0.5 | 0 | 1 | 1 | 0.5 | 0.5 | 1 | 1 | 21.5 |
| Wang et al. (2022) | 1 | 1 | 1 | 1 | 0.5 | 0 | 0 | 1 | 0 | 0.5 | 1 | 0 | 17 |
| Gao et al. (2022) | 1 | 0.5 | 1 | 1 | 0.5 | 0 | 0 | 1 | 0 | 0.5 | 1 | 1 | 16.5 |
| Reporting rate (%) | 76.5 | 76.5 | 91.2 | 94.1 | 61.8 | 35.3 | 17.6 | 91.2 | 17.6 | 32.4 | 58.8 | 47.1 | — |

— denotes no relevant results; reporting rate = [(number of studies with compete report of entries + number of studies with partial report)/total number of included studies] × 100%

**Supplementary Table S7.　GRADE classification of the included studies for MAs/SRs.**

| Study ID | Classification of outcomes indicators | Outcomes (number of studies) | Risk of bias | Inconsistency | Indirectness | Imprecision | Publication bias | Upgrading factors | Quality of evidence |
| --- | --- | --- | --- | --- | --- | --- | --- | --- | --- |
| Le et al. (2013) | Hand dexterity | FT, PPT (≤1Hz) (5) | -1^①^ | 0 | 0 | -1^④^ | -1^⑥^ | None | Very low |
|  |  | mRS, JTT (≤1Hz) (4) | -1^①^ | 0 | 0 | 0 | -1^⑥^ | None | Low |
| Ling et al. (2017) | Hand dexterity | KT, FT, WMFT (＞1Hz) (3) | -1^①^ | 0 | 0 | -1^④^ | -1^⑥^ | None | Very low |
| Wu et al. (2022) | Upper limb motor function | FMA-UE (4) | -1^①^ | -2^③^ | 0 | -1^④^ | -1^⑥^ | None | Very low |
|  |  | Brunnstrom assessment of hand motor function (3) | -1^①^ | 0 | 0 | 0 | -1^⑥^ | None | Low |
|  | Upper limb spasticity | MAS (3) | -1^①^ | 0 | 0 | 0 | -1^⑥^ | None | Low |
|  | ADL | MBI (4) | -1^①^ | -2^③^ | 0 | -1^④^ | -1^⑥^ | None | Very low |
| Xia et al. (2022) | Upper limb motor function | FMA-UE (9) | -1^①^ | -2^③^ | 0 | -1^④^ | -1^⑥^ | None | Very low |
|  |  | FMA-UE (iTBS) (7) | -1^①^ | 0 | 0 | -1^④^ | -1^⑥^ | None | Very low |
|  |  | FMA-UE (cTBS) (2) | -1^①^ | 0 | 0 | 0 | -1^⑥^ | None | Low |
|  | Hand dexterity | ARAT (4) | -1^①^ | 0 | 0 | -1^④^ | -1^⑥^ | None | Very low |
|  | Stroke severity | NIHSS (3) | -1^①^ | -2^③^ | 0 | -1^④^ | -1^⑥^ | None | Very low |
|  | ADL | MBI (6) | -1^①^ | -1^②^ | 0 | -1^④^ | -1^⑥^ | None | Very low |
| Gao (2021) | Upper limb spasticity | MAS (3) | -1^①^ | 0 | 0 | 0 | 0 | None | Moderate |
|  | Upper limb motor function | FMA-UE (9) | -1^①^ | -1^②^ | 0 | -1^④^ | 0 | None | Very low |
|  |  | FMA-UE (rTMS) (7) | -1^①^ | 0 | 0 | 0 | 0 | None | Moderate |
|  |  | FMA-UE (TBS) (2) | -1^①^ | 0 | 0 | 0 | 0 | None | Moderate |
|  |  | FMA-UE (rTMS+TBS) (2) | -1^①^ | 0 | 0 | -1^④^ | 0 | None | Low |
|  |  | FMA-UE (ischemic stroke) (4) | -1^①^ | 0 | 0 | 0 | 0 | None | Moderate |
|  |  | FMA-UE (ischemic stroke/hemorrhagic stroke) (4) | -1^①^ | 0 | 0 | 0 | 0 | None | Moderate |
|  |  | FMA-UE (stroke) (3) | -1^①^ | 0 | 0 | -1^④^ | 0 | None | Low |
|  | ADL | BI/MBI (4) | -1^①^ | -2^③^ | 0 | -1^④^ | 0 | None | Very low |
|  |  | BI/MBI (subacute phase) (2) | -1^①^ | 0 | 0 | -1^④^ | 0 | None | Low |
|  |  | BI/MBI (non-subacute phase) (2) | -1^①^ | 0 | 0 | -1^④^ | 0 | None | Low |
|  | Cortical excitability | MEP latency (unaffected hemisphere) (3) | -1^①^ | -1^②^ | 0 | 0 | 0 | None | Low |
|  | Adverse events | Adverse events rate (6) | -1^①^ | 0 | 0 | 0 | 0 | None | Moderate |
| Hsu et al. (2012) | Cortical excitability | MT (affected hemisphere) (5) | -1^①^ | 0 | 0 | 0 | 0 | None | Moderate |
| Chen et al. (2022a) | Hand dexterity | BBT, NHPT, PPT (17) | -1^①^ | 0 | 0 | 0 | -1^⑥^ | None | Low |
|  |  | BBT, NHPT, PPT (acute phase) (5) | -1^①^ | 0 | 0 | 0 | -1^⑥^ | None | Low |
|  |  | BBT, NHPT (subacute phase) (3) | -1^①^ | 0 | 0 | 0 | -1^⑥^ | None | Low |
|  |  | BBT, NHPT (chronic phase) (8) | -1^①^ | 0 | 0 | 0 | -1^⑥^ | None | Low |
|  |  | BBT, NHPT, PPT (short-term) (5) | -1^①^ | 0 | 0 | 0 | -1^⑥^ | None | Low |
|  |  | BBT, NHPT, PPT (medium-term) (5) | -1^①^ | 0 | 0 | 0 | -1^⑥^ | None | Low |
|  | Upper limb motor function (acute phase) | FMA-UE (bilateral) (2) | -1^①^ | -2^③^ | 0 | 0 | -1^⑥^ | None | Very low |
|  |  | FMA-UE (affected side) (7) | -1^①^ | -1^②^ | 0 | 0 | -1^⑥^ | None | Very low |
|  |  | FMA-UE (unaffected side) (7) | -1^①^ | -2^③^ | 0 | 0 | -1^⑥^ | None | Very low |
|  |  | FMA-UE (severe baseline impairment) (6) | -1^①^ | -2^③^ | 0 | 0 | -1^⑥^ | None | Very low |
|  |  | FMA-UE (mild baseline impairment) (1) | -1^①^ | 0 | 0 | 0 | -1^⑥^ | None | Low |
|  |  | FMA-UE (moderate baseline impairment) (5) | -1^①^ | -1^②^ | 0 | 0 | -1^⑥^ | None | Very low |
|  |  | FMA-UE (rTMS) (9) | -1^①^ | -2^③^ | 0 | 0 | -1^⑥^ | None | Very low |
|  |  | FMA-UE (TBS) (2) | -1^①^ | -2^③^ | 0 | 0 | -1^⑥^ | None | Very low |
|  |  | FMA-UE (1Hz) (7) | -1^①^ | -2^③^ | 0 | 0 | -1^⑥^ | None | Very low |
|  |  | FMA-UE (3-10Hz) (6) | -1^①^ | -1^②^ | 0 | 0 | -1^⑥^ | None | Very low |
|  |  | FMA-UE (5 sessions) (3) | -1^①^ | 0 | 0 | 0 | -1^⑥^ | None | Low |
|  |  | FMA-UE (10 sessions) (5) | -1^①^ | -1^②^ | 0 | 0 | -1^⑥^ | None | Very low |
|  |  | FMA-UE (12-15 sessions) (2) | -1^①^ | -2^③^ | 0 | 0 | -1^⑥^ | None | Very low |
|  |  | FMA-UE (20 sessions) (1) | -1^①^ | -2^③^ | 0 | -1^④^ | -1^⑥^ | None | Very low |
|  | Upper limb motor function (subacute phase) | FMA-UE (bilateral) (2) | -1^①^ | 0 | 0 | 0 | -1^⑥^ | None | Low |
|  |  | FMA-UE (affected side) (6) | -1^①^ | -1^②^ | 0 | 0 | -1^⑥^ | None | Very low |
|  |  | FMA-UE (unaffected side) (9) | -1^①^ | -1^②^ | 0 | 0 | -1^⑥^ | None | Very low |
|  |  | FMA-UE (severe baseline impairment) (6) | -1^①^ | -1^②^ | 0 | 0 | -1^⑥^ | None | Very low |
|  |  | FMA-UE (mild baseline impairment) (3) | -1^①^ | -2^③^ | 0 | 0 | -1^⑥^ | None | Very low |
|  |  | FMA-UE (moderate baseline impairment) (4) | -1^①^ | -1^②^ | 0 | 0 | -1^⑥^ | None | Very low |
|  |  | FMA-UE (rTMS) (12) | -1^①^ | -1^②^ | 0 | 0 | -1^⑥^ | None | Very low |
|  |  | FMA-UE (TBS) (1) | -1^①^ | 0 | 0 | 0 | -1^⑥^ | None | Low |
|  |  | FMA-UE (1Hz) (9) | -1^①^ | -1^②^ | 0 | 0 | -1^⑥^ | None | Very low |
|  |  | FMA-UE (3-10Hz) (3) | -1^①^ | -2^③^ | 0 | 0 | -1^⑥^ | None | Very low |
|  |  | FMA-UE (20Hz) (2) | -1^①^ | 0 | 0 | 0 | -1^⑥^ | None | Low |
|  |  | FMA-UE (5 sessions) (1) | -1^①^ | 0 | 0 | 0 | -1^⑥^ | None | Low |
|  |  | FMA-UE (10 sessions) (9) | -1^①^ | -1^②^ | 0 | 0 | -1^⑥^ | None | Very low |
|  |  | FMA-UE (15 sessions) (1) | -1^①^ | 0 | 0 | 0 | -1^⑥^ | None | Low |
|  |  | FMA-UE (20 sessions) (1) | -1^①^ | 0 | 0 | 0 | -1^⑥^ | None | Low |
|  |  | FMA-UE (40 sessions) (2) | -1^①^ | 0 | 0 | 0 | -1^⑥^ | None | Low |
|  | Upper limb motor function (chronic phase) | FMA-UE (bilateral) (3) | -1^①^ | -1^②^ | 0 | 0 | -1^⑥^ | None | Very low |
|  |  | FMA-UE (affected side) (5) | -1^①^ | 0 | 0 | 0 | -1^⑥^ | None | Low |
|  |  | FMA-UE (unaffected side) (10) | -1^①^ | -1^②^ | 0 | 0 | -1^⑥^ | None | Very low |
|  |  | FMA-UE (severe baseline impairment) (7) | -1^①^ | 0 | 0 | 0 | -1^⑥^ | None | Low |
|  |  | FMA-UE (moderate baseline impairment) (7) | -1^①^ | -1^②^ | 0 | 0 | -1^⑥^ | None | Very low |
|  |  | FMA-UE (rTMS) (10) | -1^①^ | -1^②^ | 0 | 0 | -1^⑥^ | None | Very low |
|  |  | FMA-UE (TBS) (5) | -1^①^ | 0 | 0 | 0 | -1^⑥^ | None | Low |
|  |  | FMA-UE (1Hz) (10) | -1^①^ | 0 | 0 | 0 | -1^⑥^ | None | Low |
|  |  | FMA-UE (3-10Hz) (1) | -1^①^ | -1^②^ | 0 | 0 | -1^⑥^ | None | Very low |
|  |  | FMA-UE (10 sessions) (8) | -1^①^ | 0 | 0 | 0 | -1^⑥^ | None | Low |
|  |  | FMA-UE (15 sessions) (2) | -1^①^ | -2^③^ | 0 | 0 | -1^⑥^ | None | Very low |
|  |  | FMA-UE (16 sessions) (1) | -1^①^ | 0 | 0 | 0 | -1^⑥^ | None | Low |
|  |  | FMA-UE (18 sessions) (2) | -1^①^ | 0 | 0 | 0 | -1^⑥^ | None | Low |
|  |  | FMA-UE (20 sessions) (2) | -1^①^ | -1^②^ | 0 | 0 | -1^⑥^ | None | Very low |
|  | Upper limb motor function | FMA-UE (Follow-up: short-term) (14) | -1^①^ | 0 | 0 | 0 | -1^⑥^ | None | Low |
|  |  | FMA-UE (Follow-up: intermediate) (23) | -1^①^ | -2^③^ | 0 | 0 | -1^⑥^ | None | Very low |
|  |  | FMA-UE (follow-up: long-term) (3) | -1^①^ | -2^③^ | 0 | 0 | -1^⑥^ | None | Very low |
| Tang et al. (2022) | Upper limb motor function | FMA-UE (11) | -1^①^ | -1^②^ | 0 | -1^④^ | -1^⑥^ | None | Very low |
|  |  | FMA-UE (iTBS) (3) | -1^①^ | 0 | 0 | -1^④^ | -1^⑥^ | None | Very low |
|  |  | FMA-UE (HF-rTMS) (8) | -1^①^ | -1^②^ | 0 | -1^④^ | -1^⑥^ | None | Very low |
|  |  | FMA-UE (＜1 month) (4) | -1^①^ | -1^②^ | 0 | -1^④^ | -1^⑥^ | None | Very low |
|  |  | FMA-UE (1-3 months) (3) | -1^①^ | 0 | 0 | -1^④^ | -1^⑥^ | None | Very low |
|  |  | FMA-UE (≥3 months) (4) | -1^①^ | 0 | 0 | -1^④^ | -1^⑥^ | None | Very low |
|  | Hand strength | PF, GS (7) | -1^①^ | -1^②^ | 0 | 0 | -1^⑥^ | None | Very low |
|  |  | PF, GS (iTBS) (1) | -1^①^ | 0 | 0 | 0 | -1^⑥^ | None | Low |
|  |  | PF, GS (rTMS) (4) | -1^①^ | -1^②^ | 0 | 0 | -1^⑥^ | None | Very low |
|  |  | GS (＜1 month) (1) | -1^①^ | 0 | 0 | 0 | -1^⑥^ | None | Low |
|  |  | GS (1-3 months) (2) | -1^①^ | 0 | 0 | 0 | -1^⑥^ | None | Low |
|  |  | PF, GS (≥3 months) (2) | -1^①^ | 0 | 0 | 0 | -1^⑥^ | None | Low |
|  | Hand dexterity | ARAT, BBT, NHPT, JTT, WMFT (6) | -1^①^ | 0 | 0 | 0 | -1^⑥^ | None | Low |
|  |  | ARAT, BBT, NHPT (iTBS) (3) | -1^①^ | 0 | 0 | -1^④^ | -1^⑥^ | None | Very low |
|  |  | BBT, JTT, WMFT (rTMS) (3) | -1^①^ | -1^②^ | 0 | -1^④^ | -1^⑥^ | None | Very low |
|  |  | WMFT, JTT (1-3 months) (2) | -1^①^ | 0 | 0 | 0 | -1^⑥^ | None | Low |
|  |  | ARAT, BBT, NPHT (≥3 months) (3) | -1^①^ | -1^②^ | 0 | 0 | -1^⑥^ | None | Very low |
|  | Cortical excitability | MEP amplitude (affected hemisphere) (3) | -1^①^ | 0 | 0 | 0 | -1^⑥^ | None | Low |
|  |  | MEP amplitude (unaffected hemisphere) (2) | -1^①^ | 0 | 0 | 0 | -1^⑥^ | None | Low |
| Zhang et al. (2017) | Hand dexterity | FT (6) | -1^①^ | 0 | 0 | 0 | 0 | None | Moderate |
|  |  | ARAT, WMFT, NHPT (10) | -1^①^ | 0 | 0 | 0 | 0 | None | Moderate |
|  | Hand strength | PF, GS (11) | -1^①^ | 0 | 0 | 0 | 0 | None | Moderate |
|  | Upper limb motor function | FMA-UE (7) | -1^①^ | -1^②^ | 0 | 0 | 0 | None | Low |
|  | Cortical excitability | MEP amplitude (affected hemisphere) (4) | -1^①^ | 0 | 0 | 0 | 0 | None | Moderate |
|  |  | MEP amplitude (unaffected hemisphere) (8) | -1^①^ | 0 | 0 | 0 | 0 | None | Moderate |
|  |  | rMT (affected hemisphere) (4) | -1^①^ | -2^③^ | 0 | 0 | 0 | None | Very low |
|  |  | rMT (unaffected hemisphere) (6) | -1^①^ | -1^②^ | 0 | 0 | 0 | None | Low |
| Le et al. (2014) | Hand dexterity | PPT, PF, FT (4) | -1^①^ | 0 | 0 | 0 | -1^⑥^ | None | Low |
|  |  | mRS, JTT, WMFT (3) | -1^①^ | 0 | 0 | 0 | -1^⑥^ | None | Low |
|  | Cortical excitability | MEP amplitude (paretic Side) (3) | -1^①^ | -1^②^ | 0 | 0 | -1^⑥^ | None | Very low |
|  |  | aMT (paretic Side) (3) | -1^①^ | 0 | 0 | 0 | -1^⑥^ | None | Low |
| Alashram et al. (2021) | Upper limb spasticity | MAS (rTMS/TBS) (2) | -1^①^ | 0 | 0 | 0 | -1^⑥^ | None | Low |
|  |  | MAS (rTMS/TBS combined orther treatment) (3) | -1^①^ | 0 | 0 | 0 | -1^⑥^ | None | Low |
| He et al. (2020) | Upper limb motor function | FMA-UE (15) | -1^①^ | -1^②^ | 0 | 0 | 0 | None | Low |
|  | Hand dexterity | JTT (3) | -1^①^ | 0 | 0 | 0 | 0 | None | Moderate |
|  | Hand strength | GS (5) | -1^①^ | 0 | 0 | 0 | 0 | None | Moderate |
|  | ADL | BI/MBI (11) | -1^①^ | 0 | 0 | 0 | 0 | None | Moderate |
|  | Stroke severity | NIHSS (7) | -1^①^ | 0 | 0 | 0 | 0 | None | Moderate |
| van Lieshout et al. (2019) | Upper limb motor function | FMA-UE (<1 month) (5) | -1^①^ | 0 | 0 | -1^④^ | 0 | None | Low |
|  |  | FMA-UE (1-3 months) (3) | -1^①^ | 0 | 0 | -1^④^ | 0 | None | Low |
|  |  | FMA-UE (＞6 months) (7) | -1^①^ | -1^②^ | 0 | -1^④^ | 0 | None | Very low |
|  |  | RT (1-3 months) (1) | -1^①^ | 0 | 0 | 0 | 0 | None | Moderate |
|  |  | RT (＞6 months) (2) | -1^①^ | 0 | 0 | 0 | 0 | None | Moderate |
|  |  | RT (only rTMS) (＞6 months) (1) | -1^①^ | 0 | 0 | 0 | 0 | None | Moderate |
|  | Hand dexterity | FT (＜1 month) (2) | -1^①^ | 0 | 0 | 0 | 0 | None | Moderate |
|  |  | FT (1-3 months) (3) | -1^①^ | 0 | 0 | 0 | 0 | None | Moderate |
|  |  | FT (3-6 months) (1) | 0 | 0 | 0 | 0 | 0 | None | High |
|  |  | FT (＞6 months) (1) | -1^①^ | 0 | 0 | 0 | 0 | None | Moderate |
|  |  | FT (1 session) (1-3 months) (1) | -1^①^ | 0 | 0 | 0 | 0 | None | Moderate |
|  |  | FT (only rTMS) (1-3 months) (1) | -1^①^ | 0 | 0 | 0 | 0 | None | Moderate |
|  |  | FT (only rTMS) (3-6 months) (1) | 0 | 0 | 0 | 0 | 0 | None | High |
|  |  | FT (HF-rTMS) (3-6 months) (1) | 0 | 0 | 0 | 0 | 0 | None | High |
|  |  | FT (HF-rTMS) (＞6 months) (1) | -1^①^ | 0 | 0 | 0 | 0 | None | Moderate |
|  |  | WMFT (＜1 month) (1) | 0 | 0 | 0 | 0 | 0 | None | High |
|  |  | WMFT (1-3 months) (2) | 0 | 0 | 0 | -1^④^ | 0 | None | Moderate |
|  |  | WMFT (＞6 months) (5) | -1^①^ | -2^③^ | 0 | -1^④^ | 0 | None | Very low |
|  |  | WMFT-sec (1-3 months) (1) | 0 | 0 | 0 | 0 | 0 | None | High |
|  |  | WMFT-sec (＞6 months) (4) | -1^①^ | -1^②^ | 0 | 0 | 0 | None | Low |
|  |  | ARAT (＜1 month) (1) | -1^①^ | 0 | 0 | -1^④^ | 0 | None | Low |
|  |  | ARAT (＞6 months) (4) | -1^①^ | -2^③^ | 0 | -1^④^ | 0 | None | Very low |
|  |  | JTT (＜1 month) (1) | -1^①^ | 0 | 0 | 0 | 0 | None | Moderate |
|  |  | JTT (＞6 months) (4) | -1^①^ | 0 | 0 | 0 | 0 | None | Moderate |
|  | Hand strength | GS (1 session) (＜1 month) (1) | -1^①^ | 0 | 0 | 0 | 0 | None | Moderate |
|  |  | GS (＜1 month) (5) | -1^①^ | -2^③^ | 0 | 0 | 0 | None | Very low |
|  |  | GS (1-3 months) (1) | -1^①^ | 0 | 0 | -1^④^ | 0 | None | Low |
|  |  | GS (3-6 months) (1) | 0 | 0 | 0 | -1^④^ | 0 | None | Moderate |
|  |  | GS (＞6 months) (1) | 0 | 0 | 0 | -1^④^ | 0 | None | Moderate |
|  |  | PF (3) | -1^①^ | -1^②^ | 0 | 0 | 0 | None | Low |
|  | ICF Function domain | RT, PF (1 session) (＞6 months) (3) | -1^①^ | -1^②^ | 0 | 0 | 0 | None | Low |
|  |  | FMA-UE, GS (treatment within 1 Month and outcome at 3 months) (4) | -1^①^ | 0 | 0 | 0 | 0 | None | Moderate |
|  |  | FMA-UE, GS (2-10 sessions) (＜1 month) (7) | -1^①^ | -1^②^ | 0 | 0 | 0 | None | Low |
|  |  | FMA -UE (2-10 sessions) (1-3 months) (1) | -1^①^ | 0 | 0 | 0 | 0 | None | Moderate |
|  |  | FT (2-10 sessions) (3-6 months) (1) | 0 | 0 | 0 | 0 | 0 | None | High |
|  |  | FMA-UE, GS, PF (2-10 sessions) (≥6 months) (7) | -1^①^ | 0 | 0 | 0 | 0 | None | Moderate |
|  |  | FT, FMA-UE (11-20 sessions) (1-3 months) (2) | 0 | 0 | 0 | 0 | 0 | None | High |
|  |  | GS (11-20 sessions) (3-6 months) (1) | 0 | 0 | 0 | 0 | 0 | None | High |
|  |  | FMA-UE (11-20 sessions) (＞6 months) (2) | -1^①^ | -1^②^ | 0 | 0 | 0 | None | Low |
|  |  | FMA-UE, GS (rTMS+additional therapy) (＜1 month) (7) | -1^①^ | -2^③^ | 0 | 0 | 0 | None | Very low |
|  |  | FMA-UE, GS, FT (rTMS+additional therapy) (1-3 months) (4) | -1^①^ | 0 | 0 | 0 | 0 | None | Moderate |
|  |  | GS (rTMS+additional therapy) (3-6 months) (1) | 0 | 0 | 0 | 0 | 0 | None | High |
|  |  | FMA-UE, FT, GS, PF (rTMS+additional therapy) (＞6 months) (11) | -1^①^ | -1^②^ | 0 | 0 | 0 | None | Low |
|  |  | FMA-UE, GS (only rTMS) (＜1 month) (2) | -1^①^ | 0 | 0 | 0 | 0 | None | Moderate |
|  |  | FMA-UE, GS (HF-rTMS) (＜1 month) (5) | -1^①^ | -1^②^ | 0 | 0 | 0 | None | Low |
|  |  | FMA-UE (HF-rTMS) (1-3 months) (1) | -1^①^ | 0 | 0 | 0 | 0 | None | Moderate |
|  |  | FT, GS, FMA-UE (LF-rTMS) (＜1 month) (8) | -1^①^ | 0 | 0 | 0 | 0 | None | Moderate |
|  |  | FT, FMA-UE (LF-rTMS) (1-3 months) (4) | -1^①^ | 0 | 0 | 0 | 0 | None | Moderate |
|  |  | GS, FT (LF-rTMS) (3-6 months) (2) | 0 | 0 | 0 | 0 | 0 | None | High |
|  |  | FMA-UE, GS, FT, RT, PF (LF-rTMS) (＞6 months) (12) | -1^①^ | -1^②^ | 0 | 0 | 0 | None | Low |
|  | ICF Activity domain | WMFT, JTT, BBT (1 session) (＜1 month) (1) | 0 | 0 | 0 | 0 | 0 | None | High |
|  |  | ARAT, WMFT (1 session) (＞6 months) (2) | -1^①^ | 0 | 0 | 0 | 0 | None | Moderate |
|  |  | JTT (2-10 sessions) (＜1 month) (1) | -1^①^ | 0 | 0 | 0 | 0 | None | Moderate |
|  |  | ARAT, JTT, PF, WMFT (2-10 sessions) (＞6 months) (6) | -1^①^ | -2^③^ | 0 | 0 | 0 | None | Very low |
|  |  | JTT, ARAT, WMFT (rTMS+additional therapy) (＜1 month) (3) | -1^①^ | 0 | 0 | 0 | 0 | None | Moderate |
|  |  | WMFT, JTT (rTMS+additional therapy) (1-3 months) (2) | 0 | 0 | 0 | 0 | 0 | None | High |
|  |  | ARAT, JTT, WMFT (rTMS+additional therapy) (＞6 months) (13) | -1^①^ | -1^②^ | 0 | 0 | 0 | None | Low |
|  |  | WMFT, JTT (only rTMS) (＜1 month) (1) | 0 | 0 | 0 | 0 | 0 | None | High |
|  |  | JTT (only rTMS) (＞6 months) (1) | -1^①^ | 0 | 0 | 0 | 0 | None | Moderate |
| Chen et al. (2022b) | Hand dexterity | BBT, NHPT, PPT (6) | -1^①^ | 0 | 0 | 0 | -1^⑥^ | None | Low |
| Jiang et al. (2024) |  | MAS (5) | -1^①^ | 0 | 0 | 0 | -1^⑥^ | None | Low |
|  |  | MAS (iTBS) (4) | -1^①^ | 0 | 0 | 0 | -1^⑥^ | None | Low |
|  |  | MAS (cTBS) (1) | -1^①^ | 0 | 0 | 0 | -1^⑥^ | None | Low |
|  |  | MAS (acute/subacute phase) (2) | -1^①^ | 0 | 0 | 0 | -1^⑥^ | None | Low |
|  |  | MAS (chronic phase) (1) | -1^①^ | 0 | 0 | 0 | -1^⑥^ | None | Low |
|  |  | MAS (affected hemisphere) (3) | -1^①^ | 0 | 0 | 0 | -1^⑥^ | None | Low |
|  |  | MAS (ipsilesional cerebellum) (1) | -1^①^ | 0 | 0 | 0 | -1^⑥^ | None | Low |
|  | Hand dexterity | NHPT (5) | -1^①^ | 0 | 0 | 0 | -1^⑥^ | None | Low |
|  |  | NHPT (cTBS) (3) | -1^①^ | -1^②^ | 0 | 0 | -1^⑥^ | None | Very low |
|  |  | NHPT (iTBS) (2) | -1^①^ | 0 | 0 | 0 | -1^⑥^ | None | Low |
|  |  | NHPT (acute/subacute phase) (2) | -1^①^ | 0 | 0 | 0 | -1^⑥^ | None | Low |
|  |  | NHPT (chronic phase) (3) | -1^①^ | -1^②^ | 0 | 0 | -1^⑥^ | None | Very low |
|  |  | NHPT (unaffected hemisphere) (2) | -1^①^ | 0 | 0 | 0 | -1^⑥^ | None | Low |
|  |  | NHPT (affected hemisphere) (1) | 0 | 0 | 0 | 0 | -1^⑥^ | None | Moderate |
|  |  | NHPT (≤1 month) (4) | -1^①^ | 0 | 0 | 0 | -1^⑥^ | None | Low |
|  |  | NHPT (＞1 month) (3) | -1^①^ | 0 | 0 | 0 | -1^⑥^ | None | Low |
|  |  | ARAT (6) | -1^①^ | 0 | 0 | 0 | -1^⑥^ | None | Low |
|  |  | ARAT (iTBS) (4) | -1^①^ | 0 | 0 | 0 | -1^⑥^ | None | Low |
|  |  | ARAT (priming-iTBS) (1) | 0 | 0 | 0 | 0 | -1^⑥^ | None | Moderate |
|  |  | ARAT (cTBS) (1) | -1^①^ | 0 | 0 | -1^④^ | -1^⑥^ | None | Very low |
|  |  | ARAT (acute subacute phase) (1) | 0 | 0 | 0 | -1^④^ | -1^⑥^ | None | Low |
|  |  | ARAT (chronic phase) (5) | -1^①^ | 0 | 0 | 0 | -1^⑥^ | None | Low |
|  |  | ARAT (≤1 month) (2) | -1^①^ | 0 | 0 | -1^④^ | -1^⑥^ | None | Very low |
|  |  | ARAT (＞1 month) (2) | -1^①^ | 0 | 0 | -1^④^ | -1^⑥^ | None | Very low |
|  | Upper limb motor movement | FMA-UE (9) | -1^①^ | -2^③^ | 0 | -1^④^ | -1^⑥^ | None | Very low |
|  |  | FMA-UE (iTBS) (6) | -1^①^ | 0 | 0 | 0 | -1^⑥^ | None | Low |
|  |  | FMA-UE (priming) (1) | 0 | 0 | 0 | 0 | -1^⑥^ | None | Moderate |
|  |  | FMA-UE (cTBS) (1) | -1^①^ | 0 | 0 | 0 | -1^⑥^ | None | Low |
|  |  | FMA-UE (TBS) (1) | 0 | 0 | 0 | 0 | -1^⑥^ | None | Moderate |
|  |  | FMA-UE (acute/subacute phase) (3) | -1^①^ | 0 | 0 | -1^④^ | -1^⑥^ | None | Very low |
|  |  | FMA-UE (chronic phase) (5) | -1^①^ | 0 | 0 | 0 | -1^⑥^ | None | Low |
|  |  | FMA-UE (≤1 month) (4) | -1^①^ | 0 | 0 | 0 | -1^⑥^ | None | Low |
|  |  | FMA-UE (＞1 month) (2) | -1^①^ | 0 | 0 | -1^④^ | -1^⑥^ | None | Very low |
| Wang et al. (2022) | Upper limb spasticity | MAS (11) | -1^①^ | 0 | 0 | 0 | -1^⑥^ | None | Low |
|  |  | MAS (LF-rTMS) (6) | -1^①^ | 0 | 0 | 0 | -1^⑥^ | None | Low |
|  |  | MAS (HF-rTMS) (1) | -1^①^ | 0 | 0 | 0 | -1^⑥^ | None | Low |
|  |  | MAS (LF-rTMS+HF-rTMS) (1) | -1^①^ | 0 | 0 | 0 | -1^⑥^ | None | Low |
|  |  | MAS (iTBS) (2) | -1^①^ | 0 | 0 | 0 | -1^⑥^ | None | Low |
|  |  | MAS (cTBS) (1) | -1^①^ | 0 | 0 | 0 | -1^⑥^ | None | Low |
|  |  | MAS (unaffected hemisphere) (6) | -1^①^ | 0 | 0 | 0 | -1^⑥^ | None | Low |
|  |  | MAS (affected hemisphere) (4) | -1^①^ | 0 | 0 | 0 | -1^⑥^ | None | Low |
| Gao et al. (2022) | Hand dexterity | ARAT (5) | -1^①^ | -2^③^ | 0 | 0 | -1^⑥^ | None | Very low |
|  |  | WMFT (2) | -1^①^ | -2^③^ | 0 | 0 | -1^⑥^ | None | Very low |
|  | Upper limb spasticity | MAS (4) | -1^①^ | -2^③^ | 0 | 0 | -1^⑥^ | None | Very low |
